# Genome-Wide Analysis of *MYB* Gene Family in *Chrysanthemum ×morifolium* Provides Insights into Flower Color Regulation

**DOI:** 10.3390/plants13091221

**Published:** 2024-04-28

**Authors:** Bohao Wang, Xiaohui Wen, Boxiao Fu, Yuanyuan Wei, Xiang Song, Shuangda Li, Luyao Wang, Yanbin Wu, Yan Hong, Silan Dai

**Affiliations:** 1 Beijing Key Laboratory of Ornamental Plants Germplasm Innovation & Molecular Breeding, National Engineering Research Center for Floriculture, Beijing Laboratory of Urban and Rural Ecological Environment, Key Laboratory for Genetics and Breeding of Forest Trees and Ornamental Plants of Ministry of Education, School of Landscape Architecture, Beijing Forestry University, Beijing 100083, China; 13073727998@163.com (B.W.); 2 Zhejiang Institute of Landscape Plants and Flowers, Zhejiang Academy of Agricultural Sciences, Hangzhou 311251, China

**Keywords:** chrysanthemum, *MYB* gene family, duplicates, selection pressure, anthocyanin biosynthesis, gene expression pattern

## Abstract

MYBs constitute the second largest transcription factor (TF) superfamily in flowering plants with substantial structural and functional diversity, which have been brought into focus because they affect flower colors by regulating anthocyanin biosynthesis. Up to now, the genomic data of several *Chrysanthemum* species have been released, which provides us with abundant genomic resources for revealing the evolution of the *MYB* gene family in *Chrysanthemum* species. In the present study, comparative analyses of the *MYB* gene family in six representative species, including *C. lavandulifolium*, *C. seticuspe*, *C. ×morifolium*, *Helianthus annuus*, *Lactuca sativa*, and *Arabidopsis thaliana*, were performed. A total of 1104 MYBs, which were classified into four subfamilies and 35 lineages, were identified in the three *Chrysanthemum* species (*C. lavandulifolium*, *C. seticuspe*, and *C. ×morifolium*). We found that whole-genome duplication and tandem duplication are the main duplication mechanisms that drove the occurrence of duplicates in *CmMYBs* (particularly in the R2R3-MYB subfamily) during the evolution of the cultivated chrysanthemums. Sequence structure and selective pressure analyses of the *MYB* gene family revealed that some of R2R3-MYBs were subjected to positive selection, which are mostly located on the distal telomere segments of the chromosomes and contain motifs 7 and 8. In addition, the gene expression analysis of *CmMYBs* in different organs and at various capitulum developmental stages of *C. ×morifolium* indicated that *CmMYBS2*, *CmMYB96*, and *CmMYB109* might be the negative regulators for anthocyanin biosynthesis. Our results provide the phylogenetic context for research on the genetic and functional evolution of the *MYB* gene family in *Chrysanthemum* species and deepen our understanding of the regulatory mechanism of MYB TFs on the flower color of *C. ×morifolium*.

## 1. Introduction

Cultivated chrysanthemum (*Chrysanthemum ×morifolium* Ramat.), a perennial herb of *Chrysanthemum* spp. in Asteraceae, is of great ornamental and economic value due to its rich flower colors and floral types [1,2,3]. The worldwide cultivation history of the cultivated chrysanthemums through artificial selection after long-term and multiple hybridizations among a variety of *Chrysanthemum* species dates back over 3000 years [1,4]. Due to the complexity of hybridization in such a long history, the ancestry of the modern cultivated chrysanthemums remains uncertain, even though some wild *Chrysanthemum* species such as *C. vestitum* (2*n* = 54), *C. indicum* (2*n* = 18 or 36), *C. lavandulifolium* (2*n* = 18), and *C. nankingense* (2*n* = 18) are considered as the original cross parents of the cultivated chrysanthemums [5,6,7]. Flower color is one of the most important ornamental traits of modern chrysanthemums, which is mainly determined by carotenoids and flavonoids [8,9]. White and yellow flowers are common in wild *Chrysanthemum* species based on carotenoids. In contrast, in the cultivated chrysanthemums, flower colors vary from pink to red, orange, purple, etc. based on anthocyanins, a class of flavonoids, as the dominant pigments [10]. Revealing how the wild *Chrysanthemum* species with monotonous flower colors evolved into the colorful cultivated chrysanthemums contributes new insights into the origin of the modern cultivated chrysanthemums.

MYB transcription factors (TFs) are characterized by a highly conserved MYB DNA-binding domain. This domain usually comprises up to four imperfect repeats of 50–53 amino acids, and each repeat forms a helix–turn–helix (HTH) structure that binds to DNA and intercalates into the major groove of target DNA sequences [11,12]. The *MYB* gene family can be classified into four major types based on the number of repeats: R2R3-MYB (2R-MYB), R1R2R3-MYB (3R-MYB), R1R2R2R1/2-MYB (4R-MYB), and 1R-MYB (MYB-related proteins), of which R2R3-MYB is the largest subfamily [13]. Studies have shown that the expansion of the *MYB* gene family members is mainly caused by genome-wide doubling events and small-scale duplication [14,15,16]. Plant R2R3-MYBs experienced three major expansion events: one early in the origin of land plants from Chlorophyta, one after the divergence of spermatophytes from vascular plants, and one in the common ancestor of angiosperms before the divergence of monocots and eudicots, forming the majority of the R2R3-MYB subfamily [17]. Along with the evolution of angiosperms, a series of functionally conserved or lineage-specific MYB subfamilies arose with roles in three major plant-specific biological processes: development and cell differentiation, specialized metabolism, and biotic and abiotic stresses [18]. MYBs with repression activity usually contain one or more repression domains at the C-terminal, such as ethylene-responsive element-binding factor-associated amphiphilic repression motif (EAR), sensitive to ABA and drought 2 protein interact motif (SID), TLLLFR, and R/KLFGV [19].

Anthocyanin biosynthesis is regulated by an MBW complex consisting of MYB, basic helix–loop–helix (bHLH), and WD-repeat (WD40) families [20,21,22], of which the *MYB* gene family, the second largest TF superfamily in flowering plants, plays an important role in many plants [18,23,24]. The regulation of anthocyanin biosynthesis by MYB TFs is divided into positive and negative ways [25]. *VvMYBA1* and *VvMYBA2* in grapes (*Vitis vinifera*) [26], *MYB10* in strawberries (*Fragaria ×ananassa*) [27], *RrMYB5* and *RrMYB10* in roses (*Rosa rugosa*) [28], and *MdMYB10* and *MdMYB12* in apples (*Malus pumila*) promote anthocyanin biosynthesis [29,30]. On the contrary, anthocyanin accumulation is inhibited by *AtMYB4* in Arabidopsis (*Arabidopsis thaliana*) [31], *PhMYBx* in petunias (*Petunia hybrida*) [32], *FaMYB1* in strawberries [33], and *MdMYB16* in apples [34]. Meanwhile, the regulation of anthocyanins by MYBs is also specific in temporal and spatial patterns. For example, in moth orchid (*Phalaenopsis aphrodite*), *PeMYB2*, *PeMYB11*, and *PeMYB12* regulate the anthocyanin accumulation in sepals and side lobes, of which *PeMYB11* is involved in the formation of red spots on the base of the lip lobe [35]. In addition, MYB TFs also play an important role in the development of vegetative organs and the process of stress resistance in many plants [36,37,38]. For example, *AtMYB66* is involved in the formation of the epidermis in Arabidopsis, while *ATMYB44*, *ATMYB77*, and *ATMYB73* play an important role in the stress resistance mechanism [39,40,41]. In recent years, there have been many studies on the regulation of anthocyanin biosynthesis by MYB TFs in *C. ×morifolium*. The identified CmMYB TFs are divided into two types: positive regulators are all R2R3-MYB TFs belonging to the lineages S6 and S7, which regulate anthocyanin biosynthesis by binding to bHLH to form complexes or directly acting with the promoters of structural genes [42,43,44], while negative regulators are rarely identified, including the R3-MYB CmMYB#7 [45] and three R2R3-MYBs CmMYB012 (lineage S7), CmMYB21 (lineage S19), and CmMYB3-like (lineage S7) [46,47,48]. The above studies indicate that MYBs are functionally differentiated with the development of higher plants, therefore it is of great theoretical and practical significance to investigate the evolutionary events and expression patterns of the *MYB* gene family in different plant species. Functional studies of MYBs in *C. ×morifolium* have provided us with some insights into flower color regulation. It is not yet known, however, whether these *MYB* genes function independently or in conjunction with their duplicate genes. The evolutionary expansion of the *MYB* gene family presented in this study contributes to revealing the detailed functions of MYBs on the regulation of flower colors in *C. ×morifolium*.

With the development of RNA-sequencing (RNA-seq) technology, the increasing number of sequenced genomes in Asteraceae provides us with an opportunity to investigate the evolutionary events and functional differentiation of the *MYB* gene family in the origin and domestication of the modern cultivated chrysanthemums. Although genome-wide studies of the *MYB* gene family have been reported in many plant species [18], the evolution of the *MYB* gene family in *Chrysanthemum* species, particularly in *C.* ×*morifolium*, still remains unclear. In the present study, *MYB* gene family members in three *Chrysanthemum* species were identified. The phylogeny, sequence structure, and expansion patterns of MYBs in *C. ×morifolium* were comprehensively analyzed. Furthermore, RNA-seq data were used to analyze the expression patterns of *CmMYBs* in different organs and at various capitulum developmental stages of *C. ×morifolium*. This study not only provides the phylogenetic context for research on the genetic and functional evolution of the *MYB* gene family in *Chrysanthemum* species but also contributes to identifying new MYBs that are involved in the regulation of anthocyanin biosynthesis, which could be further applied in the molecular breeding practice of flower color modification in ornamental plants.

## 2. Materials and Methods

### 2.1. Plant Materials

The plant materials used in the present study were cultivated chrysanthemums ‘Riqie Taohong’ and ‘f23’, whose sterile seedlings were taken in 2022 from the sterile incubator of Beijing Forestry University, Beijing, China. The capitulum development of the chrysanthemums was classified into seven stages, i.e., S1 to S7, of which the anthocyanin content significantly changed (decreased in ‘Riqie Taohong’ and increased in ‘f23’) at S5, S6, and S7 with corresponding changes in the flower color phenotype (faded in ‘Riqie Taohong’ and deepened in ‘f23’). Therefore, the total RNA of ‘Riqie Taohong’ and ‘f23’ at the capitulum developmental stages S5 to S7 was extracted using an RNA Extraction Kit (Biomarker Technologies, Beijing, China) for RNA-seq. The same methods were used for different organs (root, stem, leaf, and ray florets) of ‘Riqie Taohong’. Three independent biological replicates were performed for each sample.

### 2.2. Identification of MYB Subfamily Proteins and Phylogenetic Analysis

The genomic and protein sequences of six plant species, including *C. lavandulifolium*, *C.* ×*morifolium*, *C. seticuspe*, *Lactuca sativa*, *Helianthus annuus*, and *A. thaliana*, were obtained to construct a genome database (Table 1). Protein sequences of each species for the MYB domain (Pfam: PF00249) were screened again using an HMMER search implemented in HMMER3.0 with default parameters. The amino acid sequences of the obtained MYB proteins were then used to run a genome-wide DIAMOND v2.1.0 analysis for each species with an E-value of 1 × 10^−5^ and AtMYBs were used as reference sequences [13,49,50]. Multiple sequence alignment was performed using MAFFT v7.505 with default options [51]. An evolutionary tree of the MYB proteins was then constructed using the JTT + CAT model of FastTree v2.1 with the maximum likelihood algorithm, and the Shimodaira–Hasegawa test was used to estimate the reliability of each split in the tree [52]; iTOL was finally used for graphical editing.

According to the subfamily classification of AtMYBs, the constructed evolutionary tree of MYBs was further divided into four subfamilies, including 1R-MYB, R2R3-MYB, 3R-MYB, and 4R-MYB that contain 35 lineages. The numbers of each lineage in each plant species were calculated (Table S1).

### 2.3. Synteny Analysis

Synteny blocks among *C.* ×*morifolium, C. seticuspe*, *C. makinoi*, and *C. lavandulifolium* were identified using the Ortholog module of jcvi. Interspecies synteny was identified by setting the minspan to 30 (the minimum length of synteny blocks is 30 gene pairs), and the jcvi karyotype module was used for visualization [53].

Intraspecific synteny analysis was performed using the MCScanX Wrapper module of TBtools v2.057 based on the Blast and genomic annotation files. Advanced Circos was used for visualization of the obtained synteny blocks containing *MYB* gene members [54].

### 2.4. Statistics of Duplication Events in the MYB Gene Family

The different modes of gene duplication were identified using the DupGen_finder pipeline [55], which was used to identify gene pairs corresponding to the duplication types in a species. Firstly, BLASTP was performed to search the full length of homologous protein sequences in each species [56]. Then, the DupGen_finder pipeline was employed to identify the *duplication* mode using *C. lavandulifolium* as the outgroup according to previously described methods [55,57,58,59]. Duplicated genes can be classified into segmental duplication (SD), whole-genome duplication (WGD), tandem duplication (TD), proximal duplication (PD), dispersed duplication (DSD), and transposed duplication (TRD). We defined genes in syntenic blocks on non-homologous chromosomes and those in syntenic blocks with significant relative positional differences on homologous chromosomes as SD genes [60].

### 2.5. Chromosomal Localization of MYB Genes

The chromosomal localization of *MYB* genes was obtained from the gene feature annotation file. Visualization of gene location was performed using Gene Location Visualize (Advanced) of TBtools [54].

### 2.6. Evolutionary Selection Analyses of the MYB Gene Family

Transdecoder was used for missing data imputation without reference genes. Orthofinder was used for sequence alignment between *C. indicum* and *C.* ×*morifolium*, *C. vestitum* and *C.* ×*morifolium*, *C. lavandulifolium* and *C.* ×*morifolium*, *C. seticuspe* and *C.* ×*morifolium*, *C. lavandulifolium* and *C. indicum*, *C. lavandulifolium* and *C. vestitum*, *C. lavandulifolium* and *C. makinoi*, and *C. lavandulifolium* and *C. seticuspe*, as well as for the identification of orthologous genes [61].

The non-synonymous substitution (*Ka*) and synonymous substitution (*Ks*) values of *MYB* gene pairs were respectively calculated based on the encoding sequence alignment of the selected *MYB* genes to predict the evolution of orthologous *MYB* genes and the differentiation of paralogous *MYB* genes. TBtools Simple *Ka*/*Ks* Calculator was used to calculate the *Ka/Ks* values of genes [54].

### 2.7. Expression Analysis of MYB Genes

The clean data obtained by RNA-seq were compared with the genomic sequences of *C.* ×*morifolium* using Samtools v1.18. Fragments per kilobase of transcript per million mapped reads (FPKM) values of gene expression in each sample were calculated by HISAT2 v2.1.0 and StringTie v2.2.1 with FPKM = 10^9^ × C/(N × L), where C, N, and L respectively represent the number of reads of the gene, the total number of reads, and the length of the gene. Using the cultivated chrysanthemums ‘Riqie Taohong’ and ‘f23’ with three different petal types for each, the expression patterns of *MYB* genes were further analyzed. Cluster analysis was performed using log_2_ of FPKM values.

### 2.8. qRT-PCR Analysis

The total RNA of the samples was extracted using a KK Fast Plant Total RNA Kit (Beijing Zoman Biotechnology Co., Ltd., Beijing, China). The quality of the extracted RNA was detected by 1% agarose gel electrophoresis. cDNA was synthesized using an EX RT Kit (Beijing Zoman Biotechnology Co., Ltd.). The expression abundance of anthocyanin structural genes in transgenic tobacco corolla was quantified by qRT-PCR. PCR reactions were conducted using a Mini Opticon Real-time PCR System (Bio-Rad, Hercules, CA, USA) based on the SYBR Premix ExTaq (TaKaRa, Shiga, Japan) with three replicates. *CmPP2A* was used as an endogenous control to calculate the relative expression level of target genes.

### 2.9. Co-Expression Analysis of CmMYB Genes, ABA Biosynthetic Genes, and Anthocyanin Structural Genes

Four genes that are involved in ABA biosynthesis (*ZEP*, *NCED3A*, *NCED3B*, and *NCED9*) and eight anthocyanin structural genes (*CHS1*, *CHS2*, *ANS2*, *DFR*, *F3H*, *F3′H1*, *3GT*, and *3MT1*) were identified from the transcriptomic database of cultivated chrysanthemum ‘f23’ [62,63]. *CmMYBS2*, *CmMYB96*, and *CmMYB109* were used as bait genes. CoExpNetViz was used to perform co-expression analysis with a Person’s correlation coefficient ≥ 0.6 [64]. Cytoscape was used to visualize the co-expression network of the correlated genes [65].

## 3. Results

### 3.1. Construction of Evolutionary Tree and Subfamily Classification of MYB Genes

**Figure 1:**
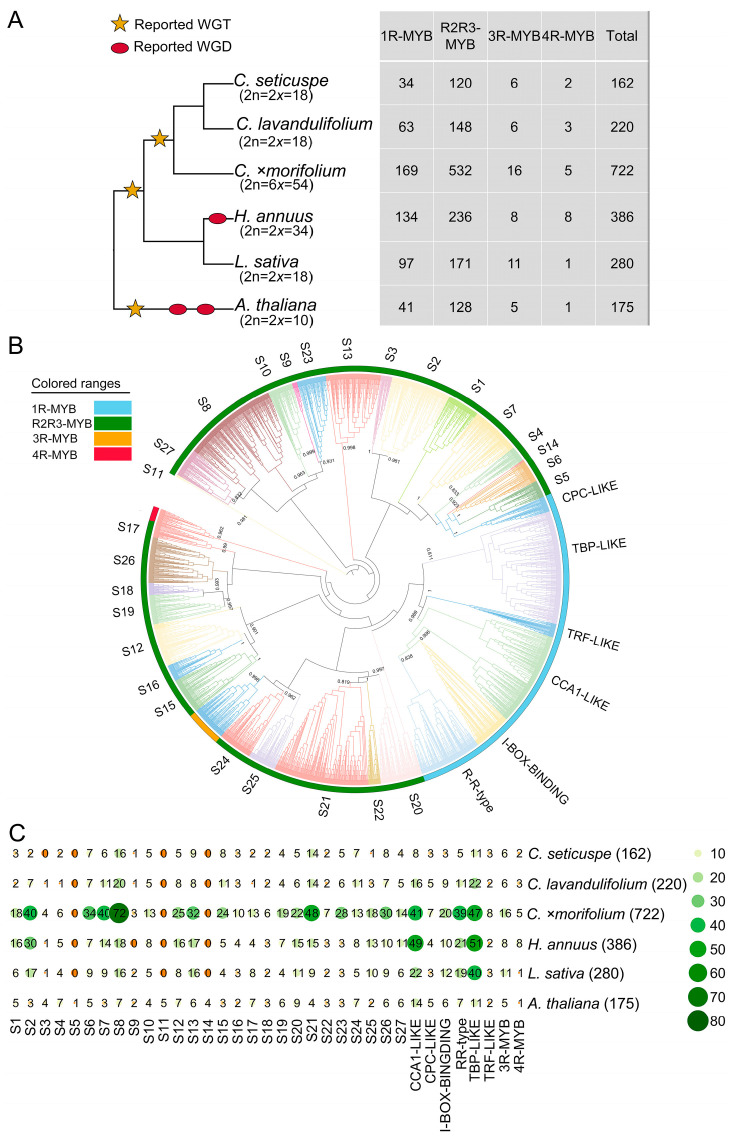
MYB protein tree and *MYB* gene numbers in six plant species. (**A**) The number of *MYB* genes identified in *C. seticuspe*, *C. lavandulifolium*, *C.* ×*morifolium*, *H. annuus*, *L. sativa*, and *A. thaliana*. (**B**) Evolutionary tree of the *MYB* gene family in the six plant species. Support values (>0.8) of each lineage calculated based on the Shimodaira–Hasegawa test are indicated. (**C**) Gene numbers in each lineage. The bigger the colored circles, the higher the expression of genes. WGT, whole-genome triplication; WGD, whole-genome duplication.

In the present study, a total of 1945 MYB family members were identified in the six plant species, including 220 in *C. lavandulifolium*, 162 in *C. seticuspe*, 722 in *C.* ×*morifolium*, 386 in *H. annuus*, 280 in *L. sativa*, and 175 in *A. thaliana* (Figure 1A). According to the classification of the *MYB* genes in *A. thaliana* [66], we divided the 1104 MYBs in *Chrysanthemum* species into four subfamilies and 35 lineages, in which R2R3-MYBs occupied ~67.3–74.1% among all members (Figure 1A,B; Table S1). The number of MYBs in the wild species *C. lavandulifolium* (220) was close to that in *C. seticuspe* (162), while in *H. annuus*, the number (386) was much higher than that in the diploid (2*n* = 2*x* = 18) wild *Chrysanthemum* species, which might be caused by a separate WGD event that occurred in the genome of sunflower after the shared whole genome triplication (WGT) event in *Chrysanthemum* species. Compared with the MYB lineages in sunflower, lettuce, and Arabidopsis, the S7 (4.7% on average), S8 (9.6% on average), S15 (7.2% on average), S21 (3.2% on average), and S23 (4.4% on average) lineages in the *Chrysanthemum* species showed a higher proportion (Figure S1). Particularly, the numbers of the *MYB* gene family in the cultivated hexaploid chrysanthemums (2*n* = 6*x* = 54) were higher than that in the wild diploid species with a ratio of 3.78:1, and the lineage S8 occupied the largest proportion (Figure 1C; Table S1). The higher number of the *MYB* gene family members in *C.* ×*morifolium* might be associated with the expansion of multiple lineages (Figure S2). A total of 32/35 (91.4%) lineages underwent an expansion event in *C.* ×*morifolium*, e.g., S2, S3, S7, S13, S17, and S25 (Figure S2). Among them, the R2R3-MYB subfamily showed a stronger expansion trend compared to those in other subfamilies (χ^2^ test, *p* < 0.001, Tables S2 and S3). In addition, some lineages such as S11 and S14 might have undergone a contraction event in Asteraceae species, while gene contraction did not appear in the lineages of 1R-MYB, 3R-MYB, and 4R-MYB (Figure 1C; Table S1). In summary, R2R3-MYBs have the highest number of expansions in *C.* ×*morifolium* compared with those in wild *Chrysanthemum* species.

### 3.2. Evolutionary Analysis of the MYB Gene Family in Chrysanthemum Species

**Figure 2:**
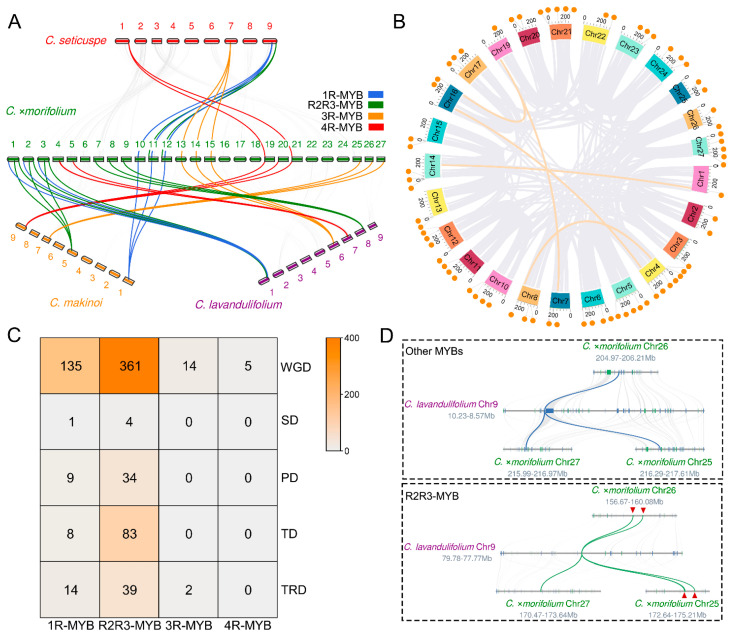
Analysis of *MYB* gene expansion between *C.* ×*morifolium* and three wild *Chrysanthemum* species. (**A**) Genome-wide synteny analysis of *C.* ×*morifolium*, *C. seticuspe*, *C. makinoi*, and *C. lavandulifolium*. Arabic numerals indicate the number of chromosomes. The colored lines indicate typical *MYB* genes that expanded through WGD in *C.* ×*morifolium*. (**B**) Location and synteny analyses of *MYB* genes in the genome of *C.* ×*morifolium*. The orange circles represent a group of tandem duplicated genes, while the orange and gray lines indicate syntenic genes produced by chromosome SD and WGD, respectively. (**C**) A heat map of the numbers of *MYB* genes produced through different expansion events in *C.* ×*morifolium*. (**D**) The typical expansion modes of *R2R3-MYB* and other *MYB* genes in *C.* ×*morifolium*. Tandem duplicated genes are marked by red triangles.

The above analyses showed that the number of *MYB* gene family members in *C.* ×*morifolium* was much higher than that in the wild *Chrysanthemum* species. Synteny analyses (the minimum length of synteny blocks is 30 gene pairs) between the three wild *Chrysanthemum* species and *C.* ×*morifolium* showed a ratio of ~1:3 for the syntenic relationship of chromosomes, which was respectively found in 517 (between *C.* ×*morifolium* and *C. lavandulifolium*), 518 (between *C.* ×*morifolium* and *C. makinoi*), and 579 (between *C.* ×*morifolium* and *C. seticuspe*) MYBs (Figure S3). This suggested that WGD occurs with the expansion of the *MYB* gene family in *C.* ×*morifolium*. However, since the ratio of the *MYB* gene family members in the wild *Chrysanthemum* species to *C.* ×*morifolium* was not strictly 1:3 (1:3.78), other expansion events of the *MYB* gene family might have occurred in *C.* ×*morifolium* in addition to WGD. Gene duplication and synteny analysis indicated that the duplication events of *MYB* genes in *C.* ×*morifolium* include WGD, SD, PD, TD, and TRD, while DSD was not detected. Most of the *MYB* genes (515/722, 71.3%) were produced by WGD. Five *MYB* genes located on Chr1, Chr4, etc. were produced by SD (Figure 2B,C). PD is another important duplication event with 6% of the identified *MYB* genes belonging to this type (Figure 2C). Additionally, 91 genes (12.6% of the total family members) located on chromosomes Chr9, Chr18, etc., were produced by TD (Figure 2B,C). Statistical analysis of the number of tandem duplicated genes in different MYB subfamilies showed that new genes were only produced by 1R-MYBs (8/91, 8.8%) and R2R3-MYBs (83/91, 91.2%). In addition, 15.6% (83/532) of the R2R3-MYB subfamily genes were produced by TD, while this proportion was 4.7% (8/169) in 1R-MYBs (Figure S4). This suggested that TD of genes in the R2R3-MYB subfamily is more frequent than those in other MYB subfamilies in *C.* ×*morifolium* (Figure 2D). Statistical analysis of the number of tandem duplicated genes in different lineages indicated that the number of tandem duplicated genes in those lineages containing more genes is relatively higher. For example, lineage S7 contained 40 genes, of which 25 genes are tandem duplicated genes; while in lineage S15, 13 of 25 genes are tandem duplicated genes. The numbers of genes and tandem duplicated genes in S7 were both higher than those in S15 (*p* < 0.01; Table S4). The remaining 55 genes (7.6% of the total number of genes) were produced by TRD in addition to the above-mentioned expansion types, which was similar to the average share of transposed genes in Arabidopsis (11%, Figure 2C) [67].

Based on the above analyses, it was speculated that SD, WGD, PD, TD, and TRD of *MYB* genes occurred with the evolution of *C.* ×*morifolium*. Most of these MYBs are produced by WGD, and TD events occur more frequently in the R2R3-MYB subfamily.

### 3.3. Sequence Structure and Selective Pressure Analyses of MYBs in C. ×morifolium

**Figure 3:**
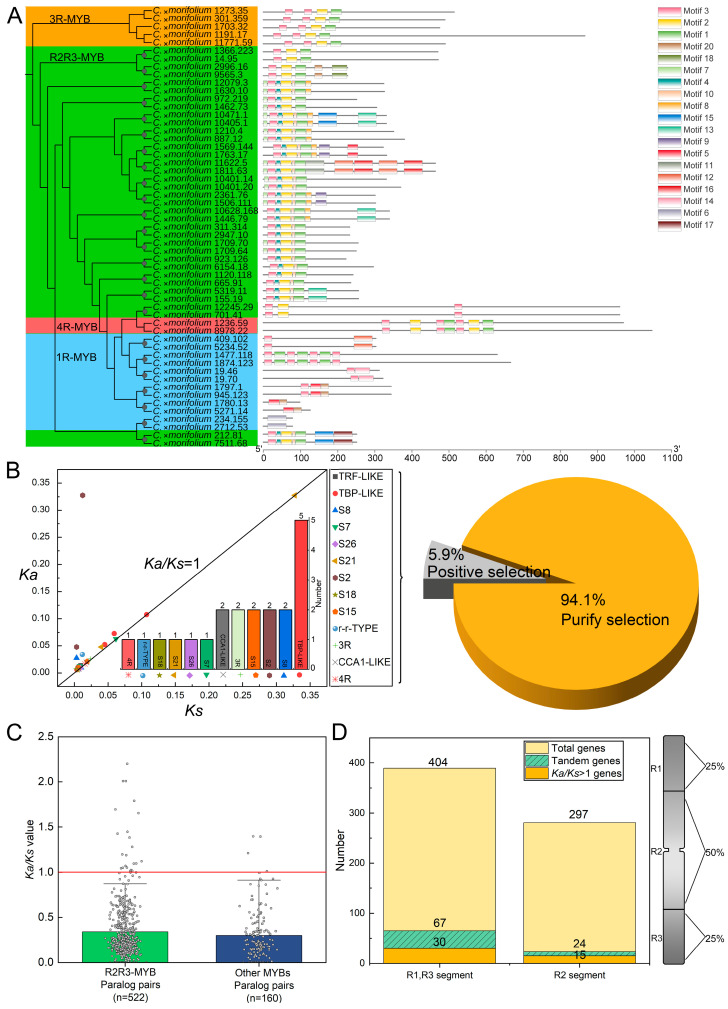
Sequence structure and selective pressure analyses of the MYB gene family in *C.* ×*morifolium*. (**A**) Sequence structure analysis of the MYB subfamily in *C. ×morifolium*. Gray circles indicate different clusters. (**B**) Selective pressure analysis of homologous MYB genes with *Ka/Ks* > 1 in *C. ×morifolium* and wild *Chrysanthemum* species. The horizontal and vertical axes represent Ks and Ka values, respectively. The *Ka/Ks* values of each lineage are marked by different symbols. Percentages of genes that are subjected to positive selection (*Ka/Ks* > 1) and purifying selection (*Ka/Ks* < 1) are shown in the pie chart. (**C**) The *Ka/Ks* values of paralogous gene pairs in the R2R3-MYB and other MYB subfamilies (1R-MYB, 3R-MYB, and 4R-MYB). n represents the sample size. (**D**) The numbers of positively selected genes, tandem duplicated genes, and all MYB genes in different segments of chromosomes. R1 and R3 are distal telomere segments, while R2 is proximal telomere segment.

The analysis of the sequence structure of *MYB* genes in *C.* ×*morifolium* showed that typical domains such as SANT/MYB, PLN03091 superfamily, PLN03212 superfamily, and REB1 superfamily existed in each *MYB* gene (Figure 3A and Figures S5–S8). Twenty conserved motifs (motif 1 to motif 20) of CmMYBs were identified by MEME, and motifs contained in different MYB subfamilies were diverse (Figure 3A). The sequence structure of 1R-MYBs was divided into six clusters (Cluster 1 to Cluster 6) which have the unique conserved motif 14 (Figure S6). Unique conserved motifs were not found in 3R-MYBs and 4R-MYBs (Figures S7 and S8). R2R3-MYBs contained 18 clusters based on the sequence structure (Cluster 1 to Cluster 18) with unique motifs of motif 12, motif 16, motif 17, motif 18, motif 19, and motif 20 (Figure S5). In R2R3-MYBs, sequences such as *C.* ×*morifolium 699.412* and *C.* ×*morifolium 5.3* with unique motif 8 and motif 11 have been shown to participate in anthocyanin biosynthesis in previous studies (Figure 3A and Figure S5).

Previous studies have proposed that gene duplication events generate two or more gene copies, and then one or all of these genes evolve under selective pressure to acquire novel gene functions that contribute to adaptation [68]. To investigate the genes that were subjected to positive selection during the evolution of *C.* ×*morifolium*, selection pressure analysis was carried out on the orthologous genes between *C.* ×*morifolium* and wild *Chrysanthemum* species. The results showed that a total of 358 *MYB* genes, which were distributed in 33 MYB lineages, were subjected to positive selection or purification in *C.* ×*morifolium* (Table S5). A total of 21 genes (5.9%) in the 12 lineages such as TBP-LIKE, S8, and CCA1-LIKE were positively selected. The number of genes in the lineage TBP-LIKE was the highest (five genes) with varied *Ka*/*Ks* values between 1.001 and 8.535 (Figure 3B and Figure S9; Table S5). A total of 337 genes (94.1%) in the 33 lineages such as r-r-TYPE and I-BOX-BINDING were purified (Figure 3B; Table S5). Those genes that were subjected to positive selection might participate in the regulation of some biological processes during the domestication of the cultivated chrysanthemums, e.g., *C.* ×*morifolium asm20_new.1011* (ortholog of *LHY* in Arabidopsis) for ABA biosynthesis in the lineage CCA1-LIKE and *C.* ×*morifolium 1111.95* (ortholog of *MYB73* in Arabidopsis) for stress resistance in the lineage S21. Some genes were positively selected in multiple orthologous comparisons. For example, *C.* ×*morifolium 485.66* (ortholog of *MYB12* in Arabidopsis), which belongs to the lineage S7, might participate in flavonoid metabolism (Figure S9; Table S5). Only a few positively selected genes were found in orthologous comparisons of wild *Chrysanthemum* species, for example, *C. lavandulifolium 0005019* (ortholog of *MYB16* in Arabidopsis) in the lineage S9 might participate in wax synthesis in plants (Table S5). Paralogous evolution analysis of the *MYB* genes that were generated by duplication events revealed 682 paralogous gene pairs. The gene numbers in 1R-MYB, R2R3-MYB, 3R-MYB, and 4R-MYB were respectively 141, 522, 15, and 4 (Figure 3C). The results of the evolutionary analysis showed that 83.3% (20/24) of the paralogous genes that were subjected to positive selection (*Ka*/*Ks* value > 1) were distributed in the R2R3-MYB subfamily, such as *C.* ×*morifolium 1742.65* and *C.* ×*morifolium 1111.119* (Table S6). Meanwhile, only four paralogous genes with *Ka*/*Ks* value > 1 were found in other MYB subfamilies (1R-MYB, 3R-MYB, and 4R-MYB), such as *C.* ×*morifolium 7475.40* and *C.* ×*morifolium 311.122* (Figure 3C; Table S6). Paralogous genes (*Ka*/*Ks* value > 1) that were subjected to purification respectively occupied 95.2% in R2R3-MYB and 97.7% in other MYB subfamilies (Table S6). The above results indicated that in *C.* ×*morifolium*, most of the paralogous *MYB* genes that were produced by duplications tended to retain their original functions, however, some other genes were subjected to positive selection, and their functions might be differentiated, particularly in the R2R3-MYB subfamily. Analysis of orthologous and paralogous *R2R3-MYB* genes showed that in addition to the characteristic MYB domains motif 1, motif 2, motif 3, motif 4, and motif 10, some domains such as motif 7 and motif 8 also existed in the sequences of the positively selected genes (Table S7).

Chromosomes were classified into three segments according to the percentage of their length, namely R1 (25% of the total chromosome length), R2 (50% of the total chromosome length), and R3 (75% of the total chromosome length). The location of chromosomes of the *MYB* gene family members in *C.* ×*morifolium* was further analyzed (Figure 3D). In the distal telomere segments of chromosomes (R1 and R3), the number of *MYB* genes was greater than that in the proximal telomere segments (R2), which follows the rule that the gene density in the proximal region is less than that in the distal region of chromosomes [69]. Additionally, 66.7% of the positively selected genes and 73.6% of the tandem duplicated genes in *C. ×morifolium* were located at the distal telomere segments. These results indicated that the evolutionary recombination events of *CmMYB* genes mainly occur at the distal telomere segments of chromosomes (Figure 3D) [69,70]. Therefore, we proposed that some of R2R3-MYBs are subjected to positive selection, which are mostly located on the distal telomere segments of the chromosomes and contain motifs 7 and 8.

### 3.4. Expression Patterns Analysis of CmMYB Genes in C. ×morifolium

**Figure 4:**
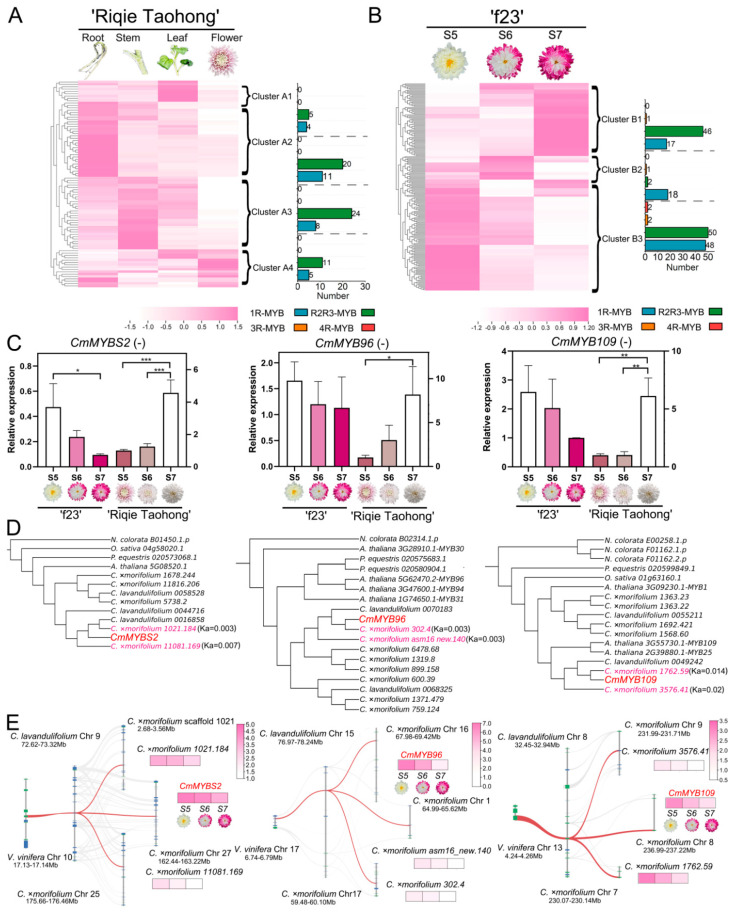
Analysis of expression patterns of *MYB* genes in *C.* ×*morifolium*. (**A**) The expression patterns of *MYB* genes in different organs of the cultivated chrysanthemum ‘Riqie Taohong’. (**B**) The expression patterns of *MYB* genes at different capitulum developmental stages of the cultivated chrysanthemum ‘f23’. S5, S6, and S7 respectively indicate the early, middle, and late stages of anthocyanin accumulation. (**C**) qRT-PCR analyses of *CmMYBS2*, *CmMYB96*, and *CmMYB109* at the capitulum developmental stages S5–S7 in the cultivated chrysanthemums ‘Riqie Taohong’ and ‘f23’. * *p* < 0.05; ** *p* < 0.01; *** *p* < 0.001. (**D**) Evolutionary analyses of *CmMYBS2*, *CmMYB96*, and *CmMYB109*. (**E**) Microsynteny visualization of *MYB* gene expansion patterns in *V. vinifera*, *C. lavandulifolium*, and *C. ×morifolium* and heat maps of the expression of different replicators. The pink squares represent the expression amount of the candidate genes at different capitulum developmental stages of the cultivated chrysanthemum ‘f23’.

Based on RNA-seq data of different organs (roots, stems, leaves, and ray florets) in the cultivated chrysanthemum ‘Riqie Taohong’ and at various capitulum developmental stages (S5, S6, and S7) in ‘f23’, the expression patterns of *CmMYB* genes were analyzed. First, a hierarchical cluster analysis was conducted on 88 MYBs (FPKM ≥ 1) in ‘Riqie Taohong’, which showed four clusters (A1 to A4) (Figure 4A). Genes in Cluster A1 were highly expressed in leaves, with 64.5% of the genes belonging to the 1R-MYB subfamily and the rest belonging to the R2R3-MYB subfamily; in addition, 88.8% of the genes were produced by WGD. Genes with high expression in roots were clustered in A2, with 80% of the genes belonging to the R2R3-MYB subfamily, and 90.6% of the genes were produced by WGD. Genes in Cluster A3 were highly expressed in stems, with 55.6% belonging to the R2R3-MYB subfamily and the rest belonging to the 1R-MYB subfamily; 80.6% of the genes were produced by WGD. Finally, 68.8% of the genes in Cluster A4 with high expression in ray florets belonged to the R2R3-MYB subfamily, and 81.3% of the genes were produced by WGD. The results also indicated that *C.* ×*morifolium 67.113* was produced by TD. The *Ka/Ks* value of *C.* ×*morifolium 485.66* (1.02) was greater than 1, indicating that this gene was subjected to positive selection. It was also found that the expression patterns of genes in the same lineage were differentiated, for example, genes in the lineage S21 were distributed both in Clusters A2 and A4. On the contrary, genes in some clusters were only distributed in a single cluster, e.g., genes in the lineages S12 and S23 were only distributed in Cluster A3 (Table S8). We also found that the expression patterns of homologous *MYB* genes were connected with the changes in protein sequences after gene expansion, i.e., the greater the *Ka* values between homologous genes, the greater the change in protein sequences and the expression patterns between homologous genes (Figure 4A; Table S8). In general, there was a strong positive correlation between expression difference and non-synonymous substitution (Figure S10; Table S8). Based on the above results, it was speculated that in *C.* ×*morifolium*, there are two main trends in the expression patterns of *MYB* genes after gene expansion: (1) some replicators are preserved without changing the original sequences, whose expression patterns are little changed; and (2) the original sequences of some other replicators are changed, resulting in a great change in their expression patterns.

Another hierarchical cluster analysis was conducted on 187 MYBs (FPKM ≥ 1) in ‘f23’, which showed three clusters (B1 to B3) (Figure 4B). Genes in Cluster B3 were highly expressed at the capitulum developmental stage S5 (early stage of anthocyanin accumulation), with 49% of the genes belonging to the R2R3-MYB subfamily and 47.1% of the genes belonging to the 1R-MYB subfamily; 89.2% of the genes were produced by WGD; the *Ka/Ks* value of *C.* ×*morifolium asm20_new.1011* (1.19) was greater than 1. Genes with high expression at S6 (middle stage of anthocyanin accumulation) were clustered in Cluster B2, with 85.7% of the genes belonging to the 1R-MYB subfamily, and 76.2% of the genes were produced by WGD. Genes in Cluster B1 were highly expressed at S7 (late stage of anthocyanin accumulation), with 71.9% of the genes belonging to the R2R3-MYB subfamily, and 81.3% of the genes were produced by WGD. Genes belonging to the R2R3-MYB subfamily occupied a large proportion in Cluster B1, suggesting that R2R3-MYB genes might be highly correlated to the positive accumulation process of anthocyanins in *C.* ×*morifolium*. Similar to the analyses of ‘Riqie Taohong’, we also found that the expression patterns of genes in the same lineage were differentiated, for example, genes in the lineage CCA1-LIKE were distributed in three clusters (Figure 4B; Table S8).

A Venn diagram was generated after comparing the expression patterns of genes in Cluster A4 (highly expressed in the capitulum of ‘Riqie Taohong’), Cluster B1 (might positively regulate anthocyanin accumulation), and B3 (might negatively regulate anthocyanin accumulation) (Figure S11). The results showed that *CmMYB73* (*C.* ×*morifolium 11562.20*), *CmMYB44* (*C.* ×*morifolium 419.299*), and *CmMYB70* (*C.* ×*morifolium 15.52*) were all highly expressed in the flower of ‘Riqie Taohong’, which might simultaneously participate in the positive regulation of anthocyanin accumulation in ‘f23’. *CmMYBS2* (*C.* ×*morifolium 901.18*), *CmMYB96* (*C.* ×*morifolium 393.191*), *CmMYB109* (*C.* ×*morifolium 965.144*), *CmTRFL6* (*C.* ×*morifolium 727.220*), and *CmMYB94* (*C.* ×*morifolium 1371.479*) were all highly expressed in the flower of ‘Riqie Taohong’ and might simultaneously participate in the negative regulation of anthocyanin accumulation in ‘f23’. The expression patterns of these eight candidate genes in the capitula of ‘Riqie Taohong’ and ‘f23’ were further analyzed by qRT-PCR.

As shown in Figure 4C, the anthocyanin content gradually decreased from the capitulum developmental stages S5 to S7 in ‘Riqie Taohong’, while in ‘f23’, an opposite result was obtained. Therefore, *CmMYBS2* was negatively correlated with anthocyanin accumulation in the two cultivated chrysanthemums, whose expression gradually decreased with the anthocyanin accumulation. Additionally, the expression patterns of *CmMYB96* and *CmMYB109* were similar to that of *CmMYBS2*, which might also negatively regulate anthocyanin accumulation. *CmMYBS2*, *CmMYB96*, and *CmMYB109* are therefore speculated as important TF genes in the anthocyanin biosynthesis of *C.* ×*morifolium*. The evolutionary events and sequence structure of these three genes were further analyzed based on published literature. The homologous analysis showed that *CmMYBS2*, an ortholog of *MYBS2* in Arabidopsis, belonged to the r-r-TYPE lineage [71]; *CmMYB96*, an ortholog of *MYB96* in Arabidopsis, belonged to lineage S1 [72]; *CmMYB109*, an ortholog of *MYB109* in Arabidopsis, belonged to lineage S22 [72]. These three genes are replicators generated by WGD in the evolution process of *C.* ×*morifolium*, whose branches on the phylogenetic tree are also expanded. The expression patterns of them were similar with small *Ka* values (0.008 on average) (Figure 4D; Figure S10). Moreover, the expression levels of these three genes were the highest among their replicators (Figure 4E). It was worth noting that the regulatory functions of *MYB96* and *MYB109* in anthocyanin accumulation have been reported [73,74], further suggesting their important roles in anthocyanin biosynthesis in *C.* ×*morifolium*.

### 3.5. Co-Expression Regulatory Network of CmMYBs

**Figure 5:**
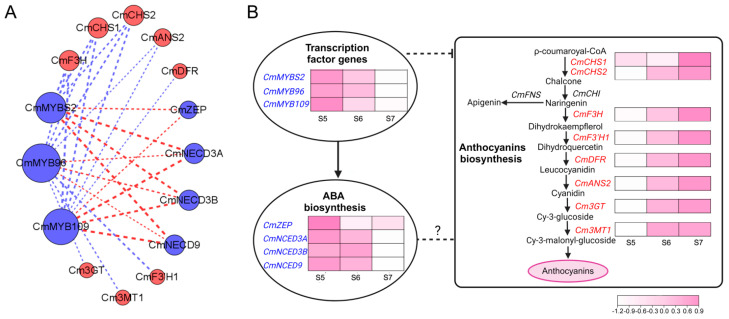
Predicted co-expression regulatory network of CmMYBs (CmMYBS2, CmMYB96, and CmMYB109) in *C.* ×*morifolium*. (**A**) Analyses of a co-expression network of CmMYBs and TFs involved in the biosynthesis of ABA and anthocyanins. The blue and red nodes respectively represent negative and positive regulators of anthocyanin biosynthesis. Co-expression values were calculated based on Pearson’s correlation coefficient ≥0.6. The dashed lines indicate a co-expression relationship with blue lines for negative correlations and red lines for positive correlations. The thicker the lines, the stronger the co-expression relationship. (**B**) A predicted network of CmMYBs on the regulation of ABA and anthocyanin biosynthesis. S5, S6, and S7 respectively indicate the early, middle, and late stages of anthocyanin accumulation in the capitulum of the cultivated chrysanthemum ‘f23’.

In previous studies, *CmMYBS2*, *CmMYB96*, and *CmMYB109* seemed to play a role in ABA biosynthesis [71,75,76]. Co-expression analyses of the identified *CmMYB* genes, anthocyanin structural genes, and ABA biosynthetic genes were further performed in *C.* ×*morifolium* based on the capitulum transcriptome of ‘f23’. *CmMYBS2*, *CmMYB96*, and *CmMYB109* were used as bait genes. It turned out that eight anthocyanin structural genes, namely chalcone synthase 1 (*CHS1*), *CHS2*, anthocyanin synthase 2 (*ANS2*), dihydroflavonol 4-hydroxylase (*DFR*), flavanone 3-hydroxylase (*F3H*), flavonoid 3′-hydroxylase 1 (*F3’H1*), anthocyanidin 3-*O*-galactosyltransferase (*3GT*), and flavonoid-3-*O*-glucosyltransferase 1 (*3MT1*), as well as four ABA biosynthetic genes, namely zeaxanthin epoxidase (*ZEP*), nine-cis-epoxycarotenoid dioxygenase 3A (*NCED3A*), *NCED3B*, and *NCED9*, were co-expressed with the three *CmMYB* genes (Figure 5A). In addition, 97 *MYB* genes might also co-express with the three *CmMYB* genes during anthocyanin accumulation in *C.* ×*morifolium* (Figure S12). These results further suggested that *CmMYBS2*, *CmMYB96*, and *CmMYB109* might play an important role in regulating anthocyanin accumulation in *C.* ×*morifolium*.

Taken together, a predicted regulatory model of anthocyanin accumulation by *CmMYBS2*, *CmMYB96*, and *CmMYB109* in *C.* ×*morifolium* was proposed (Figure 5B). These three candidate genes might regulate anthocyanin accumulation by regulating the expression of *ZEP*, *NCED3A*, *NCED3B*, and *NCED9* or by directly regulating the expression of anthocyanin structural genes. However, the negative regulatory mechanism of *CmMYBS2*, *CmMYB96*, and *CmMYB109* in anthocyanin accumulation in *C.* ×*morifolium* remains to be studied further.

## 4. Discussion

### 4.1. Expansion of the MYB Gene Family in C. ×morifolium

Previous studies have reported that the *MYB* gene family is one of the most abundant gene families in angiosperms, including 198 *MYB* genes in Arabidopsis [66], 197 in rice (*Oryza sativa*) [77], 127 in tomato (*Solanum lycopersicum*) [78], 324 in *C. nankingense* [79], and 244 in soybean (*Glycine max*) [80]. There can be dramatic differences in the gene numbers of the same gene family across plants due to the frequency of WGD events shared in angiosperms and lineage-specific expansion [81,82]. In our study, we identified and phylogenetically characterized 1104 MYBs in *Chrysanthemum* species, of which the number of MYBs in *C. ×morifolium* (722) is higher than that in other wild *Chrysanthemum* species (Table S9). Previous studies suggested that the gene numbers in specific gene families are influenced by ploidy and genome size [83]. The genome size of most of the wild *Chrysanthemum* species is ~2 Gb; we identified 220 MYBs in *C. lavandulifolium*, which is close to 162 MYBs in *C. seticuspe*. The high number of CmMYBs might be due to the 8.15 Gb genome size of the complex hexaploid cultivated chrysanthemums [60]. After gene duplication, the duplicates might undergo gene gain and loss events to serve as novelties in plants [84,85]. We also found a lack of lineages S5, S11, and S14 in Asteraceae species. In Arabidopsis, the lineage S11 genes (*MYB34*, *MYB51*, and *MYB122*) regulate the synthesis of an indole group of glucosinolates distributed in cruciferous plants [86], while MYB66 (WEREWOLF) plays an important role in root epidermal morphogenesis in the lineage S14 [87]. The absence of MYB66 of lineage S14 in poplar (*Populus tomentosa*) is associated with the loss of epidermal cell function in its roots [15]. In *C. nankingense*, the loss of this lineage was also found [79]. These studies indicate that the absence of lineages S5, S11, and S14 in the Asteraceae family might be related to certain traits that differ from other angiosperms, which needs to be further verified by functional studies. Meanwhile, we also discovered expanded lineages such as S2, S7, and S21 in *Chrysanthemum* species with MYBs that are involved in plant stress resistance, flavonol biosynthesis, and other biological processes [40,88,89]. Although plant genomic data could supply us with relatively comprehensive information on gene families, it is also hindered to some extent. Further studies with more high-quality genome data remain to be performed to investigate the expansion of the *MYB* gene family in *Chrysanthemum* species, particularly in *C. ×morifolium*.

### 4.2. Two Major Duplication Mechanisms of the MYB Gene Family in C. ×morifolium

Plant genomes have an abundance of duplicate genes that contribute to adaptation and trait modification during their evolution and cultivation history [16]. More and more plant genomic data could provide us with opportunities to know about the evolution of major gene families that facilitate plant novelties [85]. Our results showed a high number of MYBs in the *C. ×morifolium* genome, indicating that duplicated *MYB* genes might have played a role in the evolution of *C. ×morifolium*. To comprehensively investigate the functions of CmMYBs, the duplication mechanisms of *MYB* genes in *C. ×morifolium* were investigated in our study. The mechanisms of gene duplication include polyploidization (WGD), TD, SD, TRD, and retroduplication [16]. We found WGD and TD are the two major mechanisms that drive duplication in *CmMYB* genes. Polyploidization has been considered an extreme mechanism of gene duplication that leads to an increase in gene numbers and gene divergence in the plant genome [90]. As for the frequency of WGD events in Asteraceae [91], it is not surprising that WGD is the major mechanism of duplication in *CmMYB* genes (similar to Arabidopsis). Three WGDs have driven the majority of duplicated genes, which account for approximately 60% of duplicates in Arabidopsis [92]. However, WGD is not the only mechanism that generates duplicates in the gene family [16]. TD is another important duplication mechanism caused by subgenomic duplication events [93]. Tandem duplicates were found to account for 91 of the total *CmMYB* genes. Considering the multiple cross-species processes during the domestication of the chrysanthemums, duplications driven by TD mechanisms might be involved in the trait plasticity of *C. ×morifolium*.

### 4.3. Retention and Function Divergence of CmMYBs

The retained genes might evolve to have neofunctional, subfunctional, or non-functional [16]. Non-functional duplicates, which are also called pseudogenes, are not always deleted and are usually identified by their similar annotated genes and the presence of disabling mutations [94]. The expression pattern of genes is the most fundamental and intuitive way to explore this issue. We analyzed the expression patterns of *MYB* genes in different tissues and at different capitulum developmental stages of *C. ×morifolium* (accompanied by the accumulation of anthocyanins). The expression and functions of duplicates might be influenced by the duplication mechanism [95,96]. We also found that during the expansion process of the *MYB* gene family in *C. ×morifolium*, there is a strong positive correlation between the expression differences of homologous genes and non-synonymous substitution values, i.e., the greater the expression differences of homologous genes, the greater the non-synonymous substitution values, which is consistent with the expression changes in duplicated genes in Arabidopsis [97]. Through preliminary transcriptome screening, we obtained eight candidate genes that might be involved in the anthocyanin accumulation in *C. ×morifolium* flowers. qRT-PCR was further used to verify the expression patterns of those genes at different capitulum developmental stages with the anthocyanin biosynthesis. As a result, the expression levels of *CmMYBS2*, *CmMYB96*, and *CmMYB109* are all negatively correlated with the flower color phenotypes in both of the cultivated chrysanthemums, indicating that these three TF genes are negative regulators of anthocyanin biosynthesis. In addition, all of the three TF genes seem to play a role in the ABA-synthesis-mediated process. In Arabidopsis, MYBS1 and MYBS2 play antagonistic roles in controlling sugar signaling balance and participate in glucose-induced ABA biosynthesis [71]. The MYB96 protein can serve as a TF mediating the interaction between the biological clock and ABA signaling [98] and can regulate drought stress response by integrating ABA and auxin signals [99]. MYB109 negatively regulates stomatal closure under osmotic stress in Arabidopsis, stress-induced genes, and genes involved in ABA biosynthesis [76]. Based on the above results, we proposed that CmMYBS2, CmMYB96, and CmMYB109, key candidate CmMYBs in *C. ×morifolium*, might negatively regulate anthocyanin biosynthesis by affecting the synthetic process of ABA [100,101] or by directly acting with anthocyanin structural genes. The detailed functions of these three TFs remain to be investigated in the future.

## 5. Conclusions

Whole-genome duplication and tandem duplication are the main duplication mechanisms that drove the occurrence of duplicates in CmMYBs (particularly in the R2R3-MYB subfamily) during the evolution of the cultivated chrysanthemums, and some of R2R3-MYBs were subjected to positive selection, which are mostly located on the distal telomere segments of the chromosomes. *CmMYBS2*, *CmMYB96*, and *CmMYB109* might be the negative regulators for anthocyanin biosynthesis.

## Figures and Tables

**Table 1:** Genomic source of the six plant species (website accessed on 22 February 2024).

Species	Genomic Source
*C. lavandulifolium*	https://www.ncbi.nlm.nih.gov (PRJNA681093)
*C. seticuspe*	https://plantgarden.jp/
*C. ×morifolium*	https://doi.org/10.6084/m9.figshare.21655364.v2
*L. sativa*	https://www.ncbi.nlm.nih.gov (PRJNA173551)
*H. annuus*	https://www.ncbi.nlm.nih.gov (PRJNA345532)
*A. thaliana*	https://www.arabidopsis.org

## Data Availability

Data are contained within this article.

## Supplementary Materials

The following supporting information can be downloaded at: https://www.mdpi.com/article/10.3390/plants13091221/s1, Figure S1: The proportions of representative lineages in Asteraceae species and *Arabidopsis thaliana*, Figure S2: Boxplot of the ratio of *MYB* genes between *C.* ×*morifolium* and wild *Chrysanthemum* species in each lineage, Figure S3: Statistics of syntenic *MYB* gene numbers between *C.* ×*morifolium* and wild *Chrysanthemum* species, Figure S4: Number and proportion of tandem duplicated genes in R2R3-MYB and 1R-MYB in *C.* ×*morifolium*, Figure S5: Sequence structure analysis of the R2R3-MYB subfamily, Figure S6: Sequence structure analysis of the 1R-MYB subfamily, Figure S7: Sequence structure analysis of the 3R-MYB subfamily, Figure S8: Sequence structure analysis of the 4R-MYB subfamily, Figure S9: Comparative analysis of homologous *MYB* genes that were subjected to positive selection between *Chrysanthemum* species, Figure S10: Correlations of expression patterns between homologous genes and *Ka* values in the *MYB* gene family, Figure S11: The expression of candidate genes that may regulate anthocyanin biosynthesis in *C.* ×*morifolium*, Figure S12: Co-expression networks of *CmMYBS2*, *CmMYB96*, and *CmMYB109* in *C.* ×*morifolium*; Table S1: Quantitative analysis of the *MYB* gene family members in Asteraceae species and *Arabidopsis thaliana*, Table S2: The numbers of *MYB* genes between *C. ×morifolium* and wild *Chrysanthemum* species in the chi-square test, Table S3: Results of the chi-square test, Table S4: Pearson correlation coefficient between the numbers of genes and tandem duplicated genes in each MYB lineage (*n* = 13), Table S5: Homologous *Ka/Ks* gene pairs in the *MYB* gene family among *Chrysanthemum* species, Table S6: The *Ka/Ks* gene pairs of duplicated genes in the *MYB* gene family among *Chrysanthemum* species, Table S7: Statistics on the number of motifs in the sequences of the positively selected genes, Table S8: Classification and generation methods of gene lineages with different expression patterns, Table S9: The number of the *MYB* gene families in different plant genomes.
