# Supplementary material for: Genome-Wide Analysis of MYB Gene Family in Chrysanthemum ×morifolium Provides Insights into Flower Color Regulation
Source: Plants (Basel). 2024 Apr 28;13(9):1221. doi: 10.3390/plants13091221 (PMC11085527; doi:10.3390/plants13091221)
Supplement: Supplementary file 1 [file plants-13-01221-s001.zip › plants-2956044-supplementary Figures.pdf]

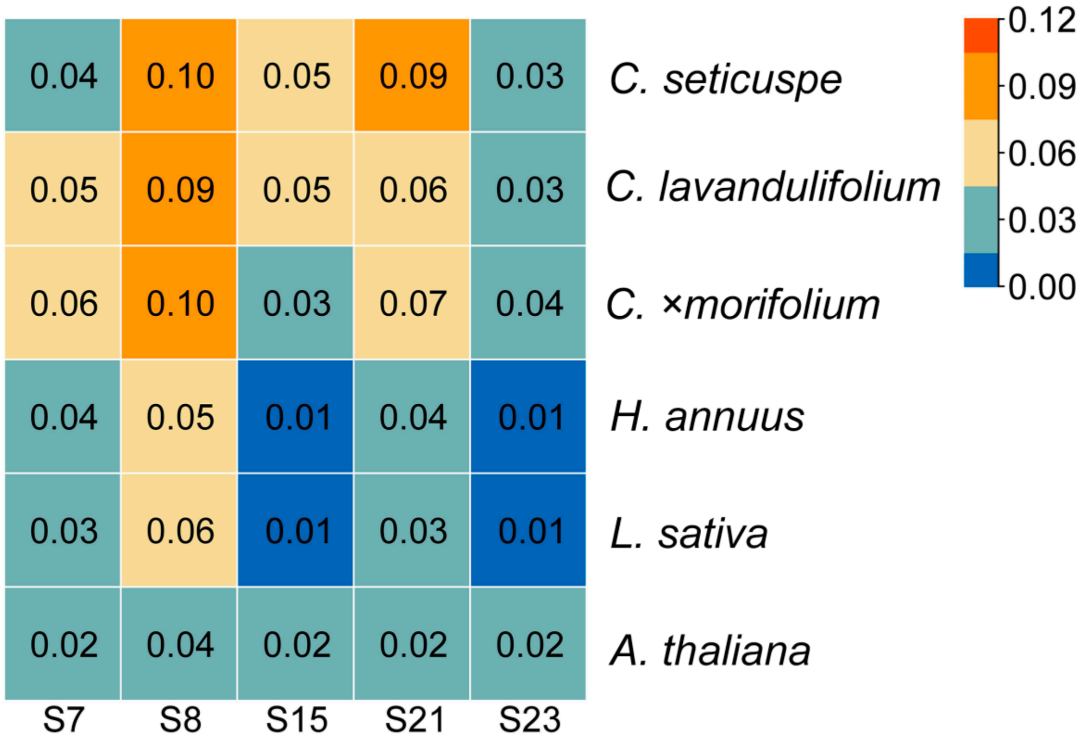

Figure S1. The proportions of representative lineages in Asteraceae species and *Arabidopsis thaliana*. The rows and columns respectively indicate different species and lineages.

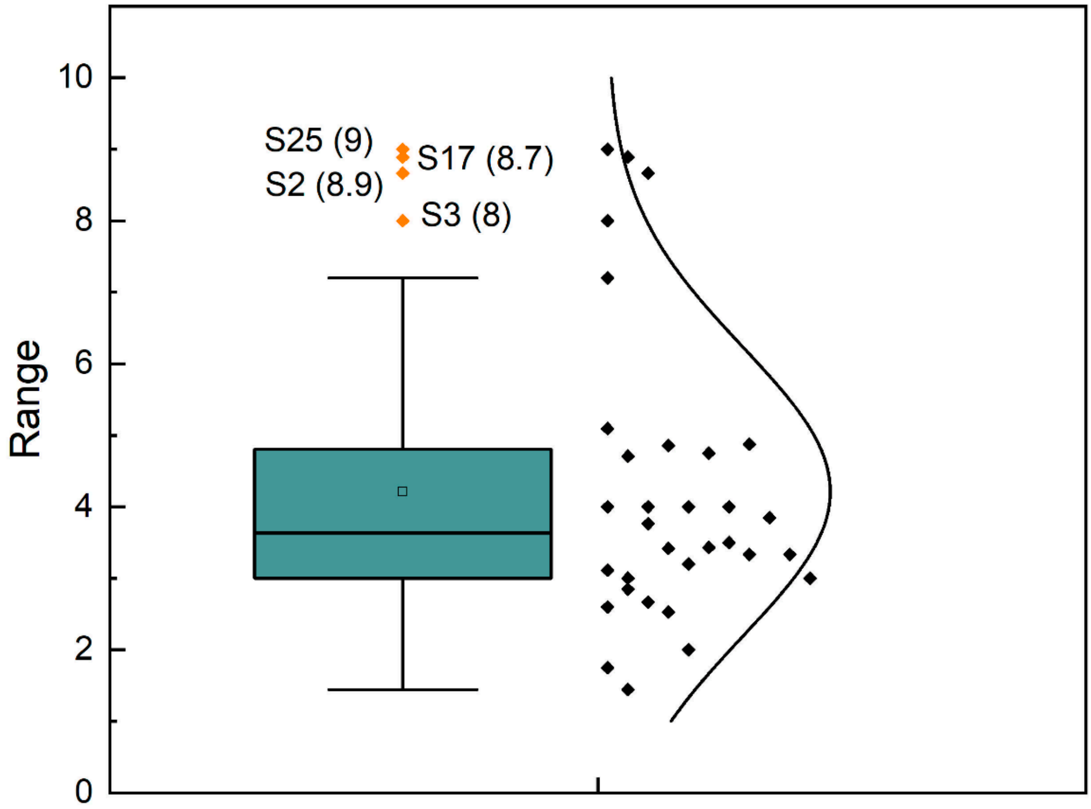

Figure S2. Boxplot of the ratio of *MYB* genes between *C.* ×*morifolium* and wild *Chrysanthemum* species in each lineage. Obvious outliers are marked in yellow.

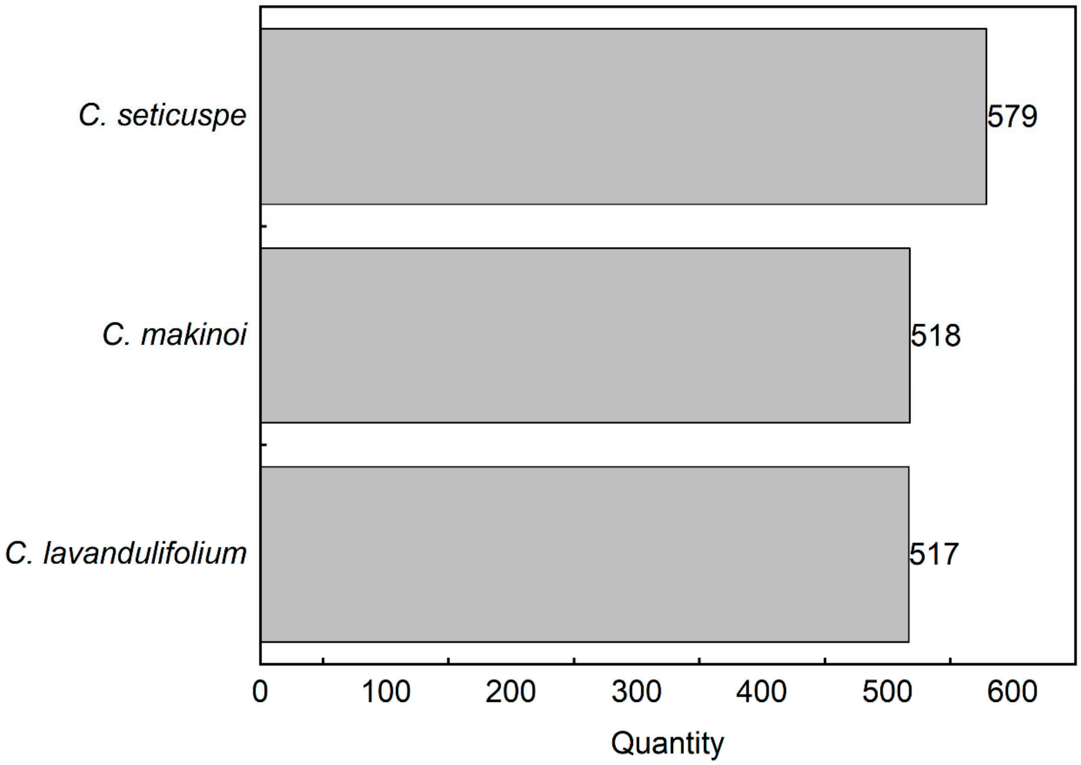

Figure S3. Statistics of syntenic *MYB* gene numbers between *C.* ×*morifolium* and wild *Chrysanthemum* species.

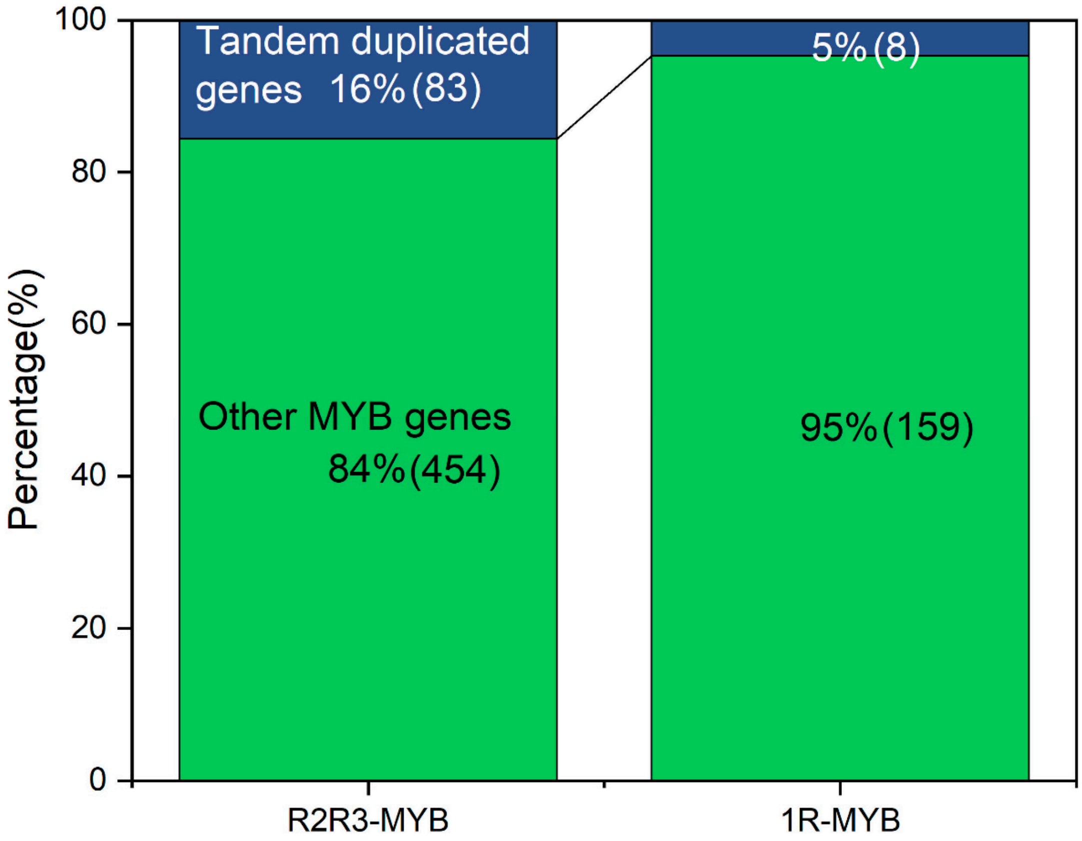

Figure S4. Number and proportion of tandem duplicated genes in R2R3-MYB and 1R-MYB in *C.* ×*morifolium*.

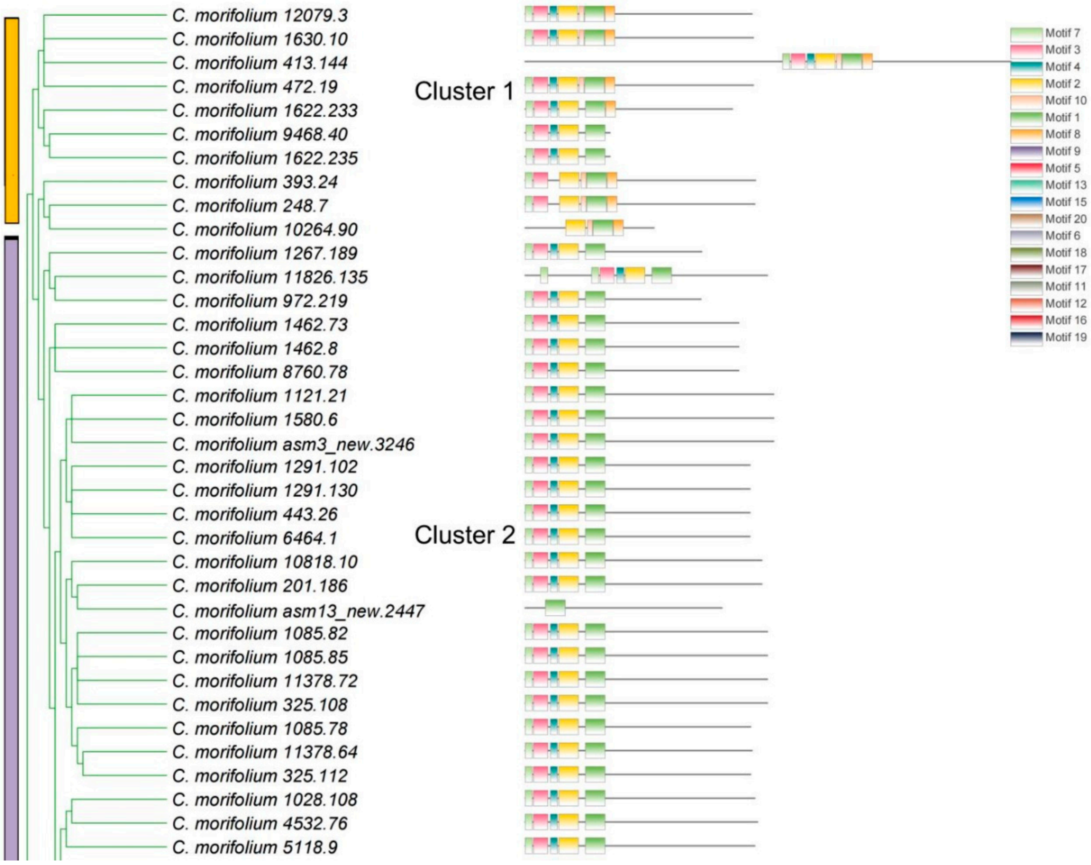

C. morifolium 12079.3
C. morifolium 1630.10
C. morifolium 413.144
C. morifolium 472.19
C. morifolium 1622.233
C. morifolium 9468.40
C. morifolium 1622.235
C. morifolium 393.24
C. morifolium 248.7
C. morifolium 10264.90
C. morifolium 1267.189
C. morifolium 11826.135
C. morifolium 972.219
C. morifolium 1462.73
C. morifolium 1462.8
C. morifolium 8760.78
C. morifolium 1121.21
C. morifolium 1580.6
C. morifolium asm3_new.3246
C. morifolium 1291.102
C. morifolium 1291.130
C. morifolium 443.26
C. morifolium 6464.1
C. morifolium 10818.10
C. morifolium 201.186
C. morifolium asm13_new.2447
C. morifolium 1085.82
C. morifolium 1085.85
C. morifolium 11378.72
C. morifolium 325.108
C. morifolium 1085.78
C. morifolium 11378.64
C. morifolium 325.112
C. morifolium 1028.108
C. morifolium 4532.76
C. morifolium 5118.9
Cluster 1
Cluster 2
Motif 7
Motif 3
Motif 4
Motif 2
Motif 10
Motif 1
Motif 8
Motif 9
Motif 5
Motif 13
Motif 15
Motif 20
Motif 6
Motif 18
Motif 17
Motif 11
Motif 12
Motif 16
Motif 19

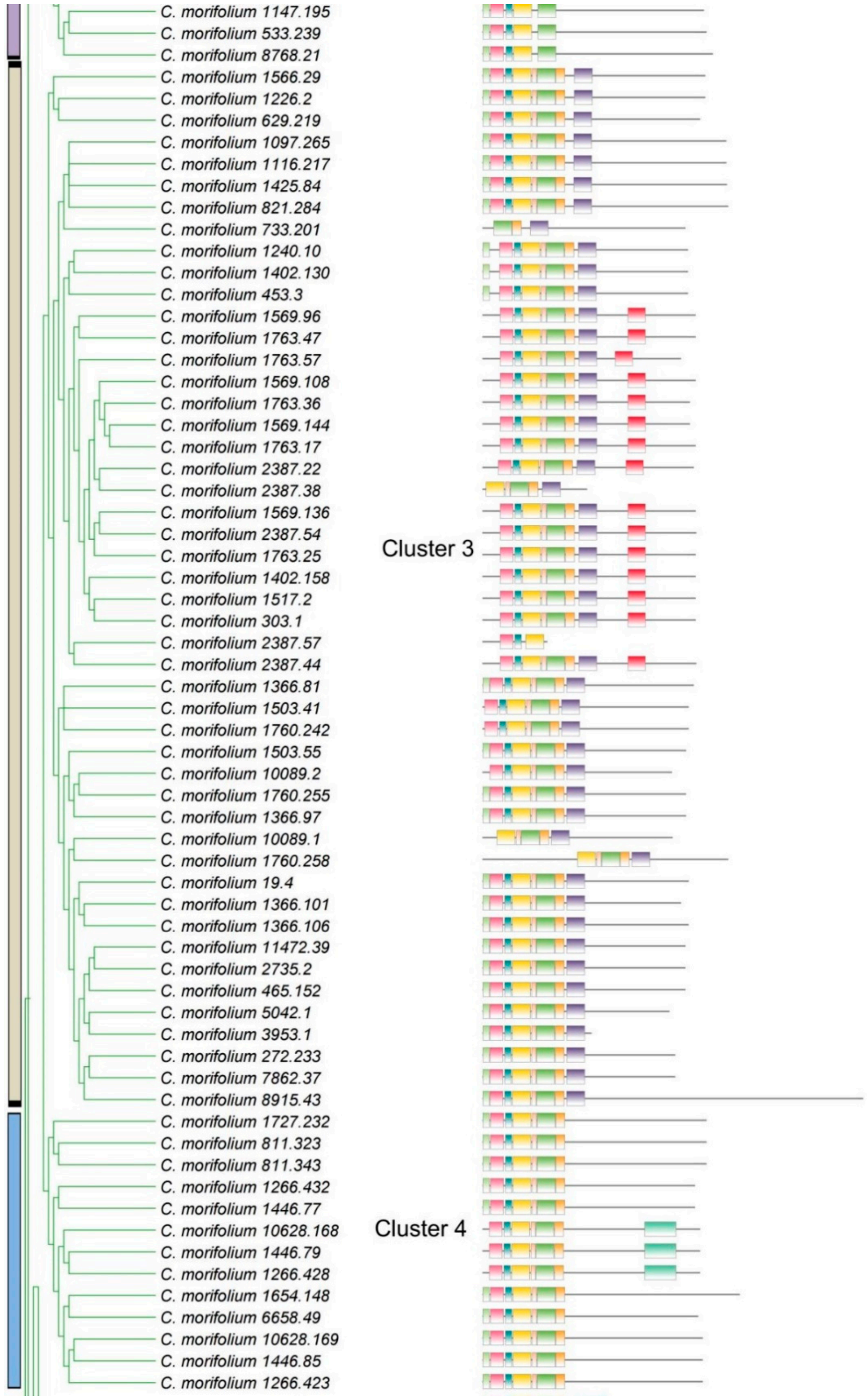

C. morifolium 1147.195
C. morifolium 533.239
C. morifolium 8768.21
C. morifolium 1566.29
C. morifolium 1226.2
C. morifolium 629.219
C. morifolium 1097.265
C. morifolium 1116.217
C. morifolium 1425.84
C. morifolium 821.284
C. morifolium 733.201
C. morifolium 1240.10
C. morifolium 1402.130
C. morifolium 453.3
C. morifolium 1569.96
C. morifolium 1763.47
C. morifolium 1763.57
C. morifolium 1569.108
C. morifolium 1763.36
C. morifolium 1569.144
C. morifolium 1763.17
C. morifolium 2387.22
C. morifolium 2387.38
C. morifolium 1569.136
C. morifolium 2387.54
C. morifolium 1763.25
C. morifolium 1402.158
C. morifolium 1517.2
C. morifolium 303.1
C. morifolium 2387.57
C. morifolium 2387.44
C. morifolium 1366.81
C. morifolium 1503.41
C. morifolium 1760.242
C. morifolium 1503.55
C. morifolium 10089.2
C. morifolium 1760.255
C. morifolium 1366.97
C. morifolium 10089.1
C. morifolium 1760.258
C. morifolium 19.4
C. morifolium 1366.101
C. morifolium 1366.106
C. morifolium 11472.39
C. morifolium 2735.2
C. morifolium 465.152
C. morifolium 5042.1
C. morifolium 3953.1
C. morifolium 272.233
C. morifolium 7862.37
C. morifolium 8915.43
C. morifolium 1727.232
C. morifolium 811.323
C. morifolium 811.343
C. morifolium 1266.432
C. morifolium 1446.77
C. morifolium 10628.168
C. morifolium 1446.79
C. morifolium 1266.428
C. morifolium 1654.148
C. morifolium 6658.49
C. morifolium 10628.169
C. morifolium 1446.85
C. morifolium 1266.423
Cluster 3
Cluster 4

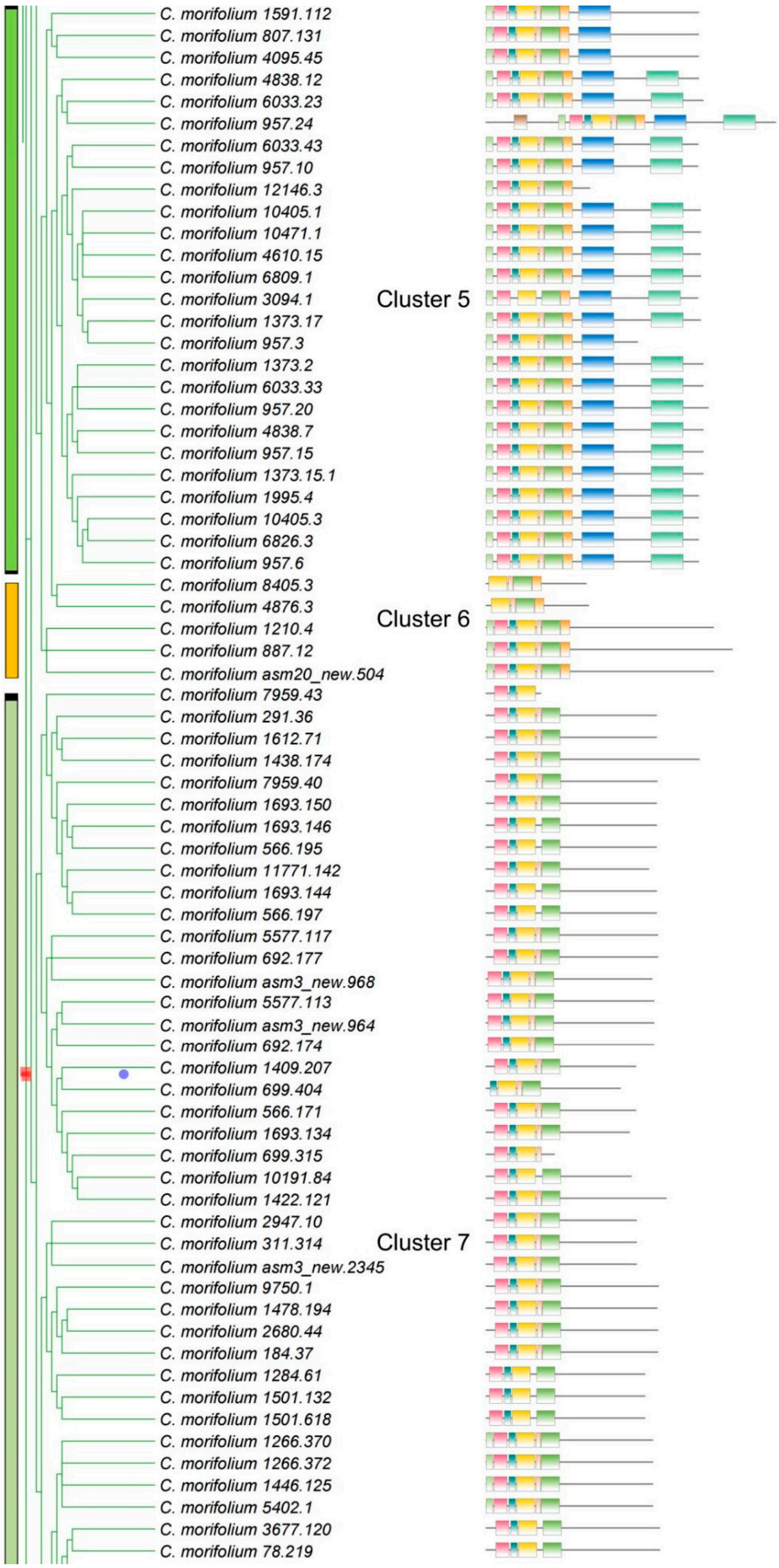
C. morifolium 1591.112
C. morifolium 807.131
C. morifolium 4095.45
C. morifolium 4838.12
C. morifolium 6033.23
C. morifolium 957.24
C. morifolium 6033.43
C. morifolium 957.10
C. morifolium 12146.3
C. morifolium 10405.1
C. morifolium 10471.1
C. morifolium 4610.15
C. morifolium 6809.1
C. morifolium 3094.1
Cluster 5
C. morifolium 1373.17
C. morifolium 957.3
C. morifolium 1373.2
C. morifolium 6033.33
C. morifolium 957.20
C. morifolium 4838.7
C. morifolium 957.15
C. morifolium 1373.15.1
C. morifolium 1995.4
C. morifolium 10405.3
C. morifolium 6826.3
C. morifolium 957.6
C. morifolium 8405.3
C. morifolium 4876.3
C. morifolium 1210.4
Cluster 6
C. morifolium 887.12
C. morifolium asm20_new.504
C. morifolium 7959.43
C. morifolium 291.36
C. morifolium 1612.71
C. morifolium 1438.174
C. morifolium 7959.40
C. morifolium 1693.150
C. morifolium 1693.146
C. morifolium 566.195
C. morifolium 11771.142
C. morifolium 1693.144
C. morifolium 566.197
C. morifolium 5577.117
C. morifolium 692.177
C. morifolium asm3_new.968
C. morifolium 5577.113
C. morifolium asm3_new.964
C. morifolium 692.174
C. morifolium 1409.207
C. morifolium 699.404
C. morifolium 566.171
C. morifolium 1693.134
C. morifolium 699.315
C. morifolium 10191.84
C. morifolium 1422.121
C. morifolium 2947.10
C. morifolium 311.314
Cluster 7
C. morifolium asm3_new.2345
C. morifolium 9750.1
C. morifolium 1478.194
C. morifolium 2680.44
C. morifolium 184.37
C. morifolium 1284.61
C. morifolium 1501.132
C. morifolium 1501.618
C. morifolium 1266.370
C. morifolium 1266.372
C. morifolium 1446.125
C. morifolium 5402.1
C. morifolium 3677.120
C. morifolium 78.219

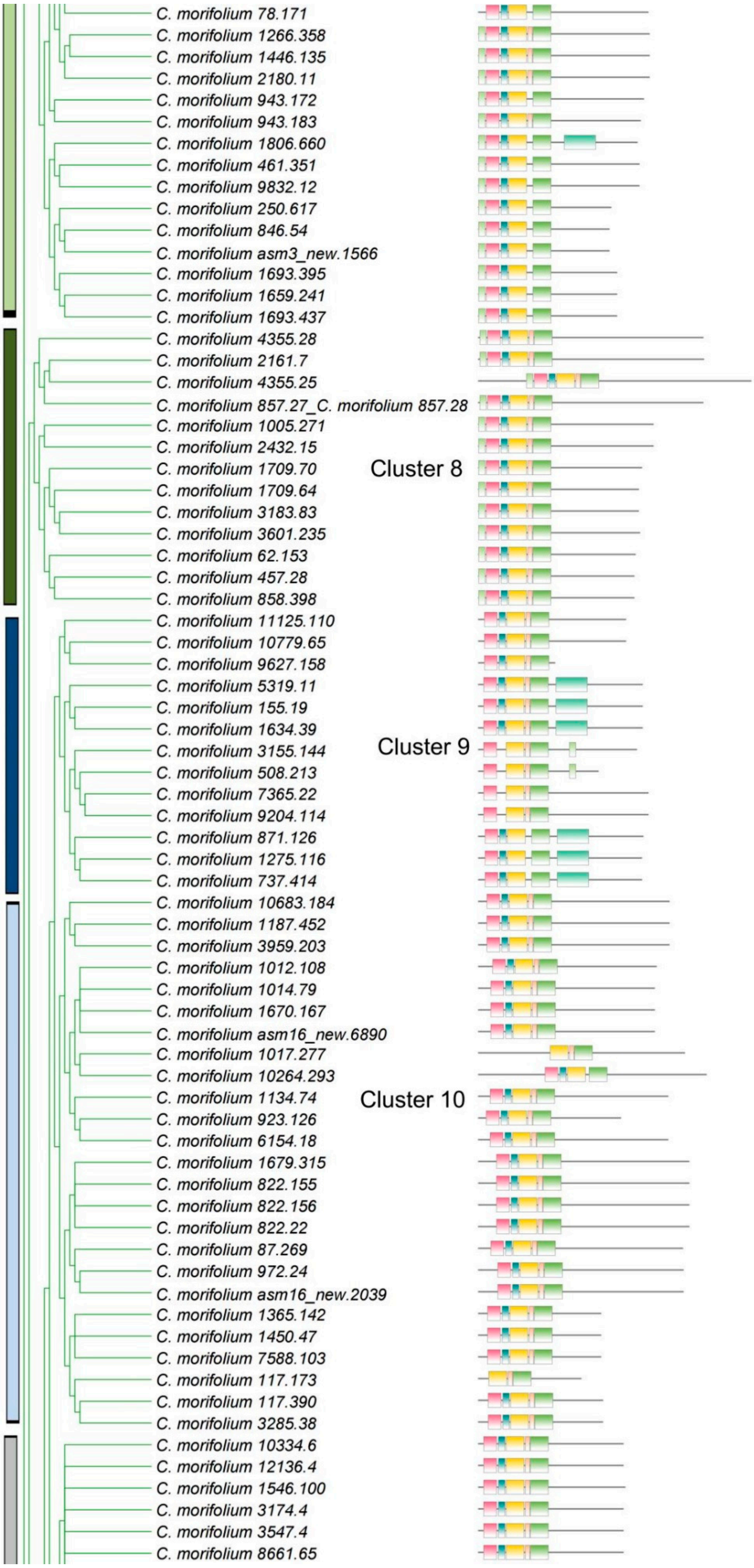
C. morifolium 78.171
C. morifolium 1266.358
C. morifolium 1446.135
C. morifolium 2180.11
C. morifolium 943.172
C. morifolium 943.183
C. morifolium 1806.660
C. morifolium 461.351
C. morifolium 9832.12
C. morifolium 250.617
C. morifolium 846.54
C. morifolium asm3_new.1566
C. morifolium 1693.395
C. morifolium 1659.241
C. morifolium 1693.437
C. morifolium 4355.28
C. morifolium 2161.7
C. morifolium 4355.25
C. morifolium 857.27_C. morifolium 857.28
C. morifolium 1005.271
C. morifolium 2432.15
C. morifolium 1709.70
Cluster 8
C. morifolium 1709.64
C. morifolium 3183.83
C. morifolium 3601.235
C. morifolium 62.153
C. morifolium 457.28
C. morifolium 858.398
C. morifolium 11125.110
C. morifolium 10779.65
C. morifolium 9627.158
C. morifolium 5319.11
C. morifolium 155.19
C. morifolium 1634.39
C. morifolium 3155.144
Cluster 9
C. morifolium 508.213
C. morifolium 7365.22
C. morifolium 9204.114
C. morifolium 871.126
C. morifolium 1275.116
C. morifolium 737.414
C. morifolium 10683.184
C. morifolium 1187.452
C. morifolium 3959.203
C. morifolium 1012.108
C. morifolium 1014.79
C. morifolium 1670.167
C. morifolium asm16_new.6890
C. morifolium 1017.277
C. morifolium 10264.293
C. morifolium 1134.74
Cluster 10
C. morifolium 923.126
C. morifolium 6154.18
C. morifolium 1679.315
C. morifolium 822.155
C. morifolium 822.156
C. morifolium 822.22
C. morifolium 87.269
C. morifolium 972.24
C. morifolium asm16_new.2039
C. morifolium 1365.142
C. morifolium 1450.47
C. morifolium 7588.103
C. morifolium 117.173
C. morifolium 117.390
C. morifolium 3285.38
C. morifolium 10334.6
C. morifolium 12136.4
C. morifolium 1546.100
C. morifolium 3174.4
C. morifolium 3547.4
C. morifolium 8661.65

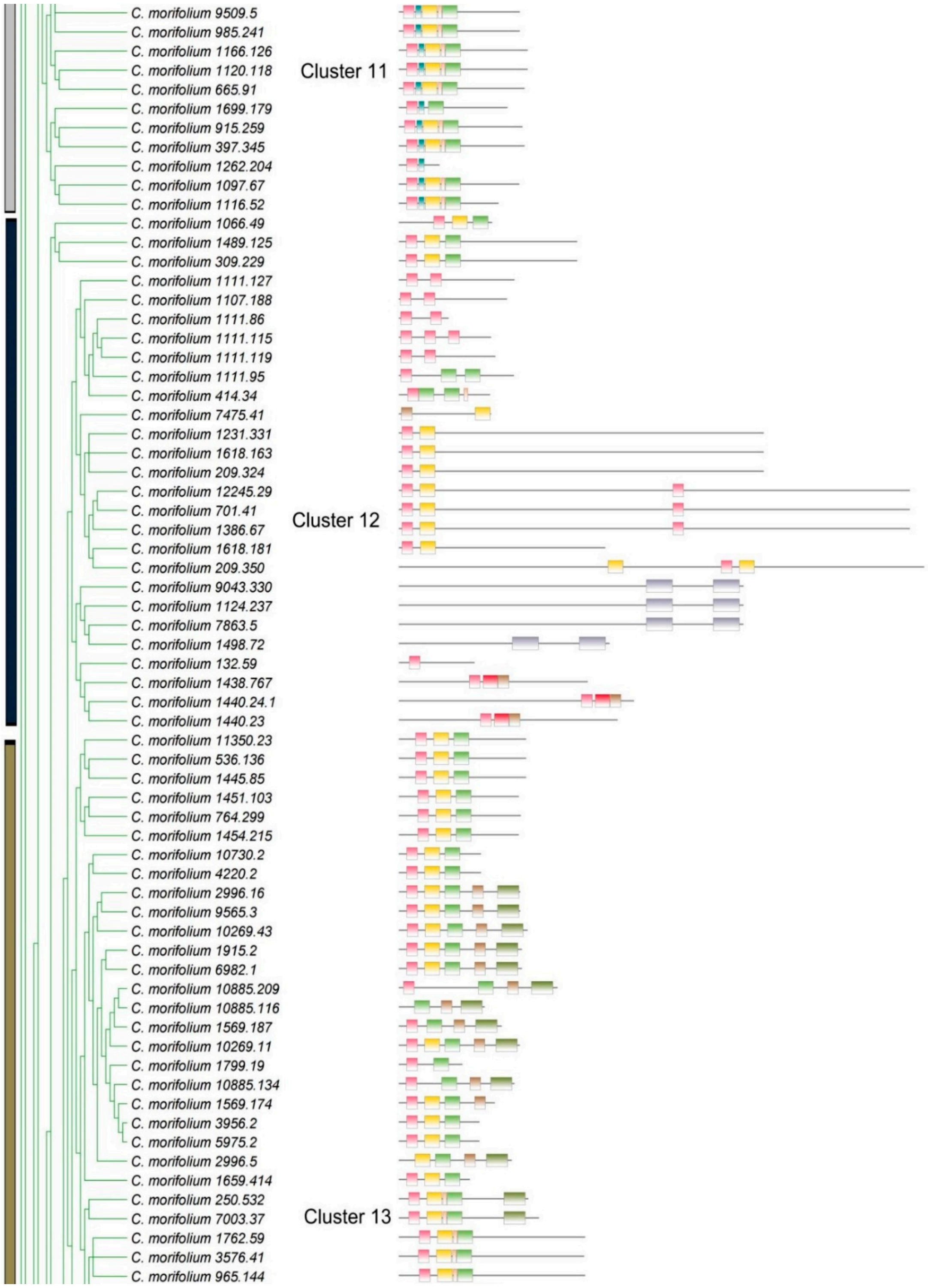
C. morifolium 9509.5
C. morifolium 985.241
C. morifolium 1166.126
C. morifolium 1120.118
C. morifolium 665.91
C. morifolium 1699.179
C. morifolium 915.259
C. morifolium 397.345
C. morifolium 1262.204
C. morifolium 1097.67
C. morifolium 1116.52
C. morifolium 1066.49
C. morifolium 1489.125
C. morifolium 309.229
C. morifolium 1111.127
C. morifolium 1107.188
C. morifolium 1111.86
C. morifolium 1111.115
C. morifolium 1111.119
C. morifolium 1111.95
C. morifolium 414.34
C. morifolium 7475.41
C. morifolium 1231.331
C. morifolium 1618.163
C. morifolium 209.324
C. morifolium 12245.29
C. morifolium 701.41
C. morifolium 1386.67
C. morifolium 1618.181
C. morifolium 209.350
C. morifolium 9043.330
C. morifolium 1124.237
C. morifolium 7863.5
C. morifolium 1498.72
C. morifolium 132.59
C. morifolium 1438.767
C. morifolium 1440.24.1
C. morifolium 1440.23
C. morifolium 11350.23
C. morifolium 536.136
C. morifolium 1445.85
C. morifolium 1451.103
C. morifolium 764.299
C. morifolium 1454.215
C. morifolium 10730.2
C. morifolium 4220.2
C. morifolium 2996.16
C. morifolium 9565.3
C. morifolium 10269.43
C. morifolium 1915.2
C. morifolium 6982.1
C. morifolium 10885.209
C. morifolium 10885.116
C. morifolium 1569.187
C. morifolium 10269.11
C. morifolium 1799.19
C. morifolium 10885.134
C. morifolium 1569.174
C. morifolium 3956.2
C. morifolium 5975.2
C. morifolium 2996.5
C. morifolium 1659.414
C. morifolium 250.532
C. morifolium 7003.37
C. morifolium 1762.59
C. morifolium 3576.41
C. morifolium 965.144
Cluster 11
Cluster 12
Cluster 13

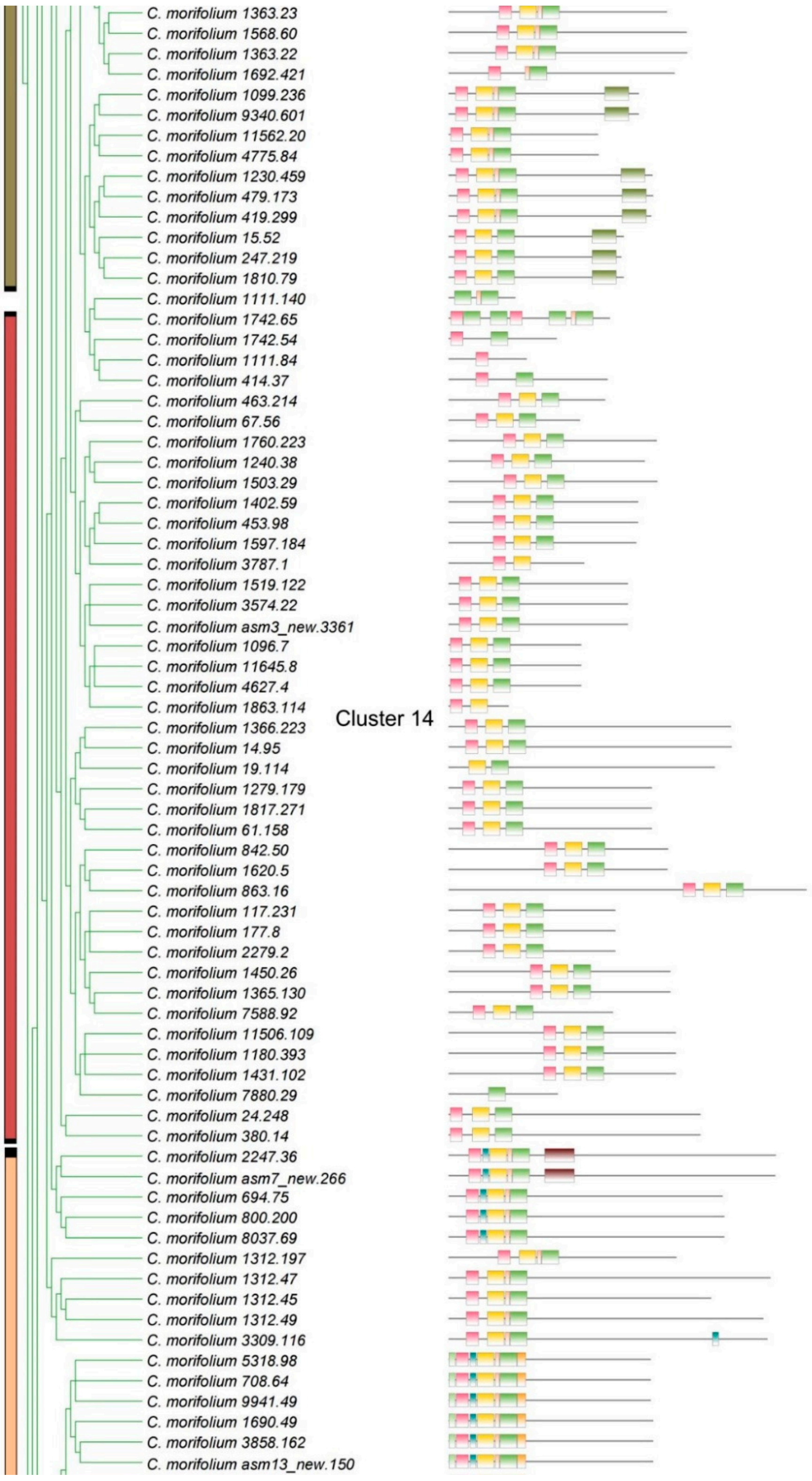

C. morifolium 1363.23
C. morifolium 1568.60
C. morifolium 1363.22
C. morifolium 1692.421
C. morifolium 1099.236
C. morifolium 9340.601
C. morifolium 11562.20
C. morifolium 4775.84
C. morifolium 1230.459
C. morifolium 479.173
C. morifolium 419.299
C. morifolium 15.52
C. morifolium 247.219
C. morifolium 1810.79
C. morifolium 1111.140
C. morifolium 1742.65
C. morifolium 1742.54
C. morifolium 1111.84
C. morifolium 414.37
C. morifolium 463.214
C. morifolium 67.56
C. morifolium 1760.223
C. morifolium 1240.38
C. morifolium 1503.29
C. morifolium 1402.59
C. morifolium 453.98
C. morifolium 1597.184
C. morifolium 3787.1
C. morifolium 1519.122
C. morifolium 3574.22
C. morifolium asm3_new.3361
C. morifolium 1096.7
C. morifolium 11645.8
C. morifolium 4627.4
C. morifolium 1863.114
Cluster 14
C. morifolium 1366.223
C. morifolium 14.95
C. morifolium 19.114
C. morifolium 1279.179
C. morifolium 1817.271
C. morifolium 61.158
C. morifolium 842.50
C. morifolium 1620.5
C. morifolium 863.16
C. morifolium 117.231
C. morifolium 177.8
C. morifolium 2279.2
C. morifolium 1450.26
C. morifolium 1365.130
C. morifolium 7588.92
C. morifolium 11506.109
C. morifolium 1180.393
C. morifolium 1431.102
C. morifolium 7880.29
C. morifolium 24.248
C. morifolium 380.14
C. morifolium 2247.36
C. morifolium asm7_new.266
C. morifolium 694.75
C. morifolium 800.200
C. morifolium 8037.69
C. morifolium 1312.197
C. morifolium 1312.47
C. morifolium 1312.45
C. morifolium 1312.49
C. morifolium 3309.116
C. morifolium 5318.98
C. morifolium 708.64
C. morifolium 9941.49
C. morifolium 1690.49
C. morifolium 3858.162
C. morifolium asm13_new.150

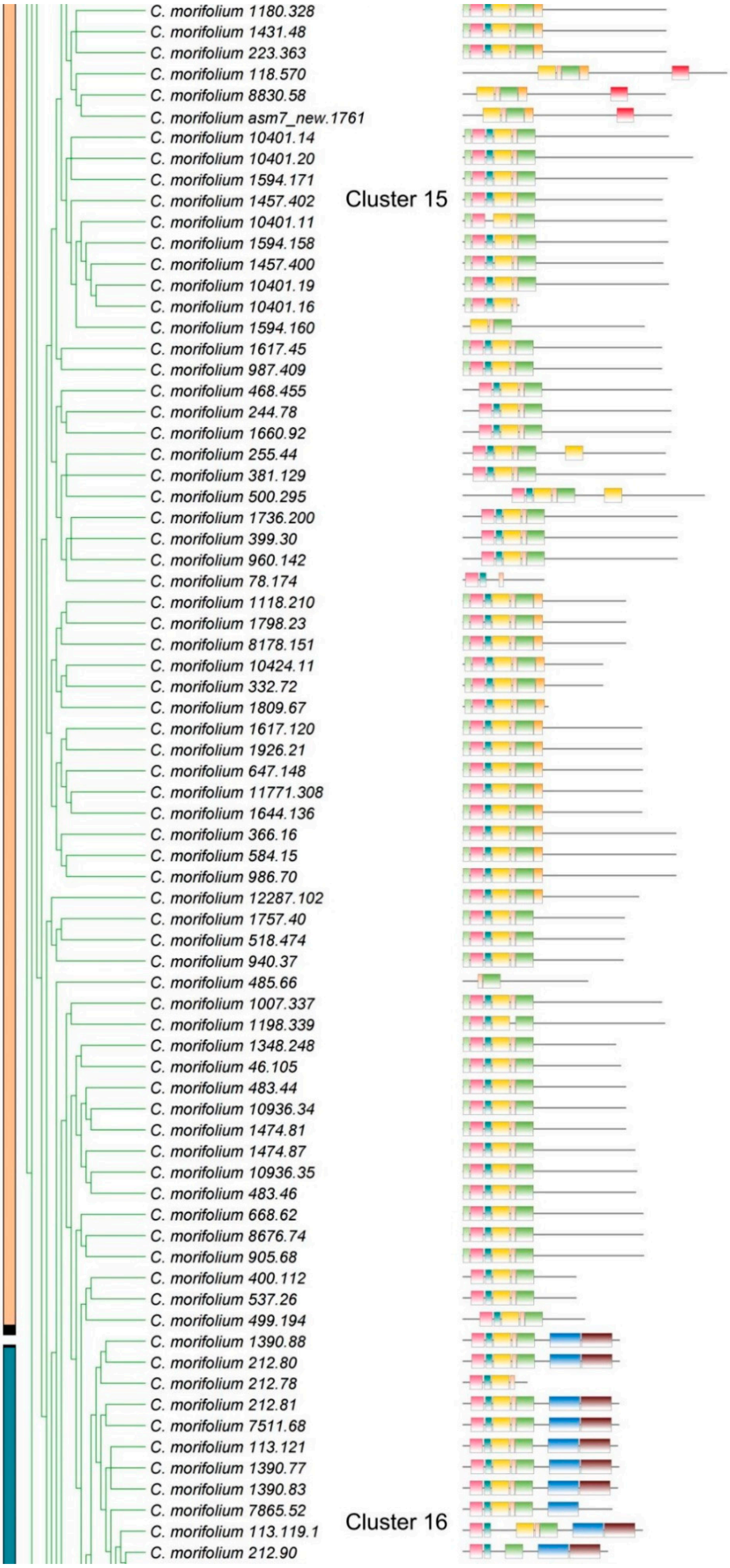
C. morifolium 1180.328
C. morifolium 1431.48
C. morifolium 223.363
C. morifolium 118.570
C. morifolium 8830.58
C. morifolium asm7_new.1761
C. morifolium 10401.14
C. morifolium 10401.20
C. morifolium 1594.171
Cluster 15
C. morifolium 1457.402
C. morifolium 10401.11
C. morifolium 1594.158
C. morifolium 1457.400
C. morifolium 10401.19
C. morifolium 10401.16
C. morifolium 1594.160
C. morifolium 1617.45
C. morifolium 987.409
C. morifolium 468.455
C. morifolium 244.78
C. morifolium 1660.92
C. morifolium 255.44
C. morifolium 381.129
C. morifolium 500.295
C. morifolium 1736.200
C. morifolium 399.30
C. morifolium 960.142
C. morifolium 78.174
C. morifolium 1118.210
C. morifolium 1798.23
C. morifolium 8178.151
C. morifolium 10424.11
C. morifolium 332.72
C. morifolium 1809.67
C. morifolium 1617.120
C. morifolium 1926.21
C. morifolium 647.148
C. morifolium 11771.308
C. morifolium 1644.136
C. morifolium 366.16
C. morifolium 584.15
C. morifolium 986.70
C. morifolium 12287.102
C. morifolium 1757.40
C. morifolium 518.474
C. morifolium 940.37
C. morifolium 485.66
C. morifolium 1007.337
C. morifolium 1198.339
C. morifolium 1348.248
C. morifolium 46.105
C. morifolium 483.44
C. morifolium 10936.34
C. morifolium 1474.81
C. morifolium 1474.87
C. morifolium 10936.35
C. morifolium 483.46
C. morifolium 668.62
C. morifolium 8676.74
C. morifolium 905.68
C. morifolium 400.112
C. morifolium 537.26
C. morifolium 499.194
C. morifolium 1390.88
C. morifolium 212.80
C. morifolium 212.78
C. morifolium 212.81
C. morifolium 7511.68
C. morifolium 113.121
C. morifolium 1390.77
C. morifolium 1390.83
C. morifolium 7865.52
Cluster 16
C. morifolium 113.119.1
C. morifolium 212.90

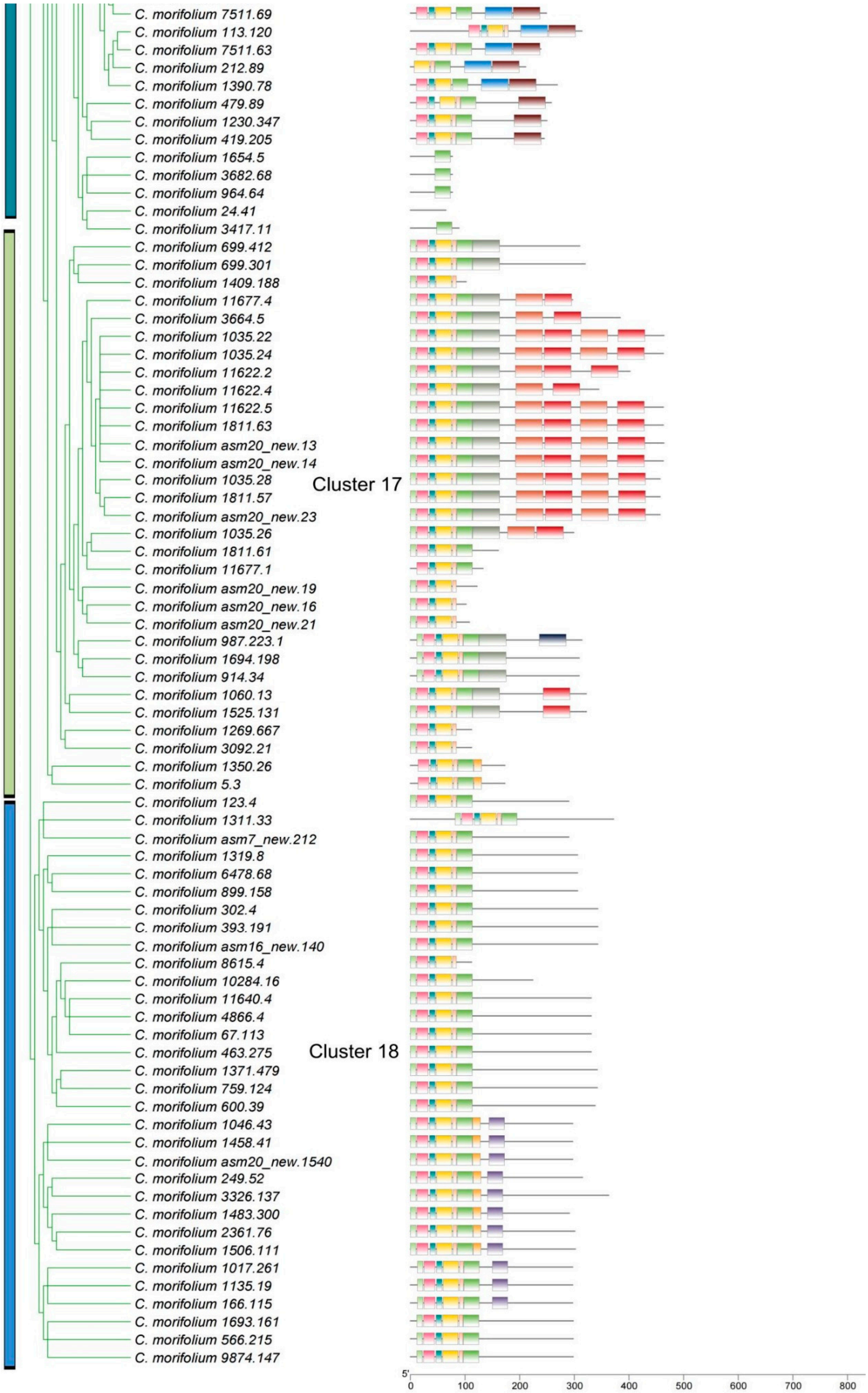

Figure S5. Sequence structure analysis of the R2R3-MYB subfamily. 19/20 conserved motifs exist in R2R3-MYBs.

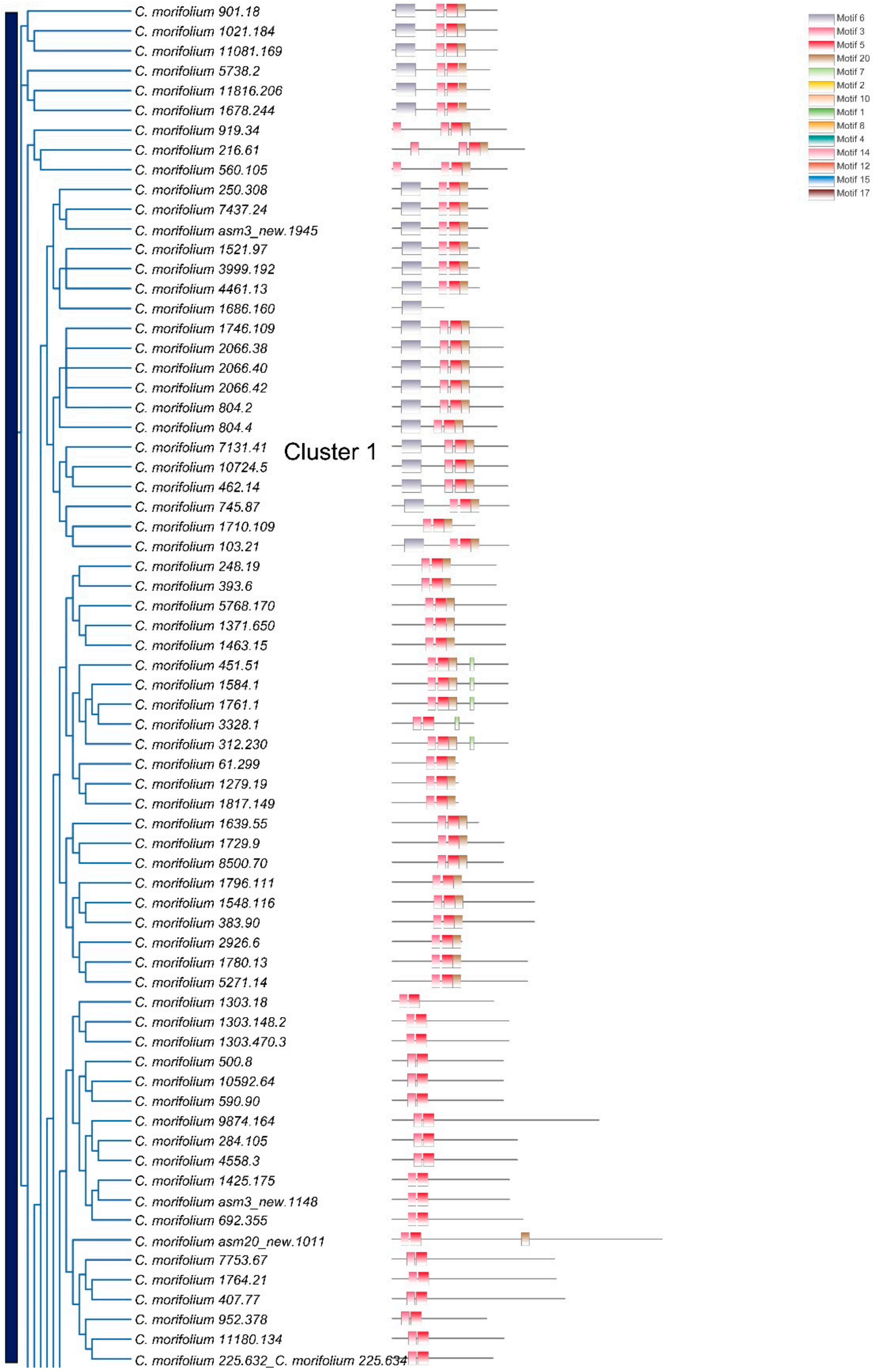
C. morifolium 901.18
C. morifolium 1021.184
C. morifolium 11081.169
C. morifolium 5738.2
C. morifolium 11816.206
C. morifolium 1678.244
C. morifolium 919.34
C. morifolium 216.61
C. morifolium 560.105
C. morifolium 250.308
C. morifolium 7437.24
C. morifolium asm3_new.1945
C. morifolium 1521.97
C. morifolium 3999.192
C. morifolium 4461.13
C. morifolium 1686.160
C. morifolium 1746.109
C. morifolium 2066.38
C. morifolium 2066.40
C. morifolium 2066.42
C. morifolium 804.2
C. morifolium 804.4
C. morifolium 7131.41
C. morifolium 10724.5
C. morifolium 462.14
C. morifolium 745.87
C. morifolium 1710.109
C. morifolium 103.21
C. morifolium 248.19
C. morifolium 393.6
C. morifolium 5768.170
C. morifolium 1371.650
C. morifolium 1463.15
C. morifolium 451.51
C. morifolium 1584.1
C. morifolium 1761.1
C. morifolium 3328.1
C. morifolium 312.230
C. morifolium 61.299
C. morifolium 1279.19
C. morifolium 1817.149
C. morifolium 1639.55
C. morifolium 1729.9
C. morifolium 8500.70
C. morifolium 1796.111
C. morifolium 1548.116
C. morifolium 383.90
C. morifolium 2926.6
C. morifolium 1780.13
C. morifolium 5271.14
C. morifolium 1303.18
C. morifolium 1303.148.2
C. morifolium 1303.470.3
C. morifolium 500.8
C. morifolium 10592.64
C. morifolium 590.90
C. morifolium 9874.164
C. morifolium 284.105
C. morifolium 4558.3
C. morifolium 1425.175
C. morifolium asm3_new.1148
C. morifolium 692.355
C. morifolium asm20_new.1011
C. morifolium 7753.67
C. morifolium 1764.21
C. morifolium 407.77
C. morifolium 952.378
C. morifolium 11180.134
C. morifolium 225.632_C. morifolium 225.634
Cluster 1
Motif 6
Motif 3
Motif 5
Motif 20
Motif 7
Motif 2
Motif 10
Motif 1
Motif 8
Motif 4
Motif 14
Motif 12
Motif 15
Motif 17

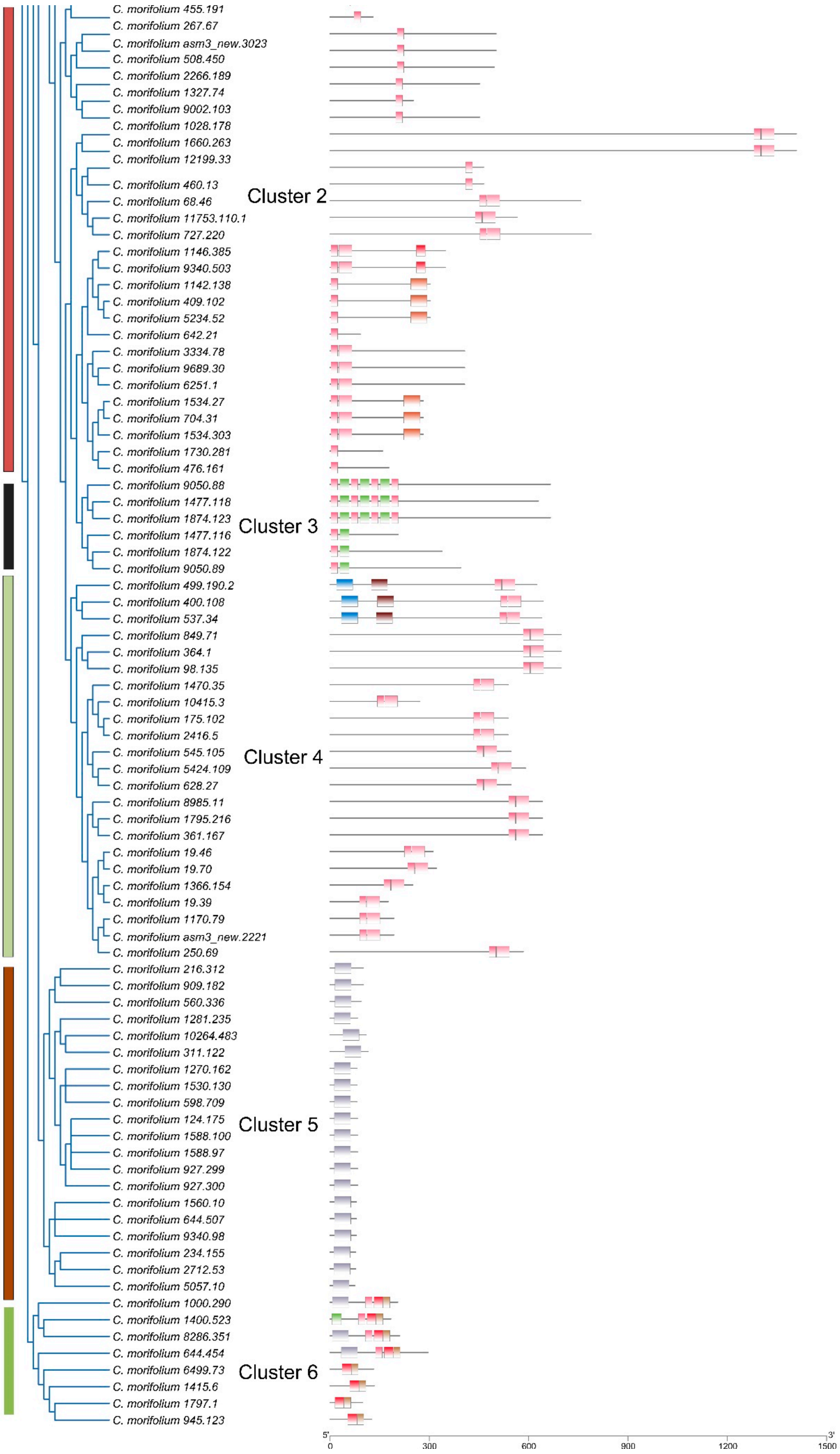

Figure S6. Sequence structure analysis of the 1R-MYB subfamily. 14/20 conserved motifs exist in 1R-MYBs.

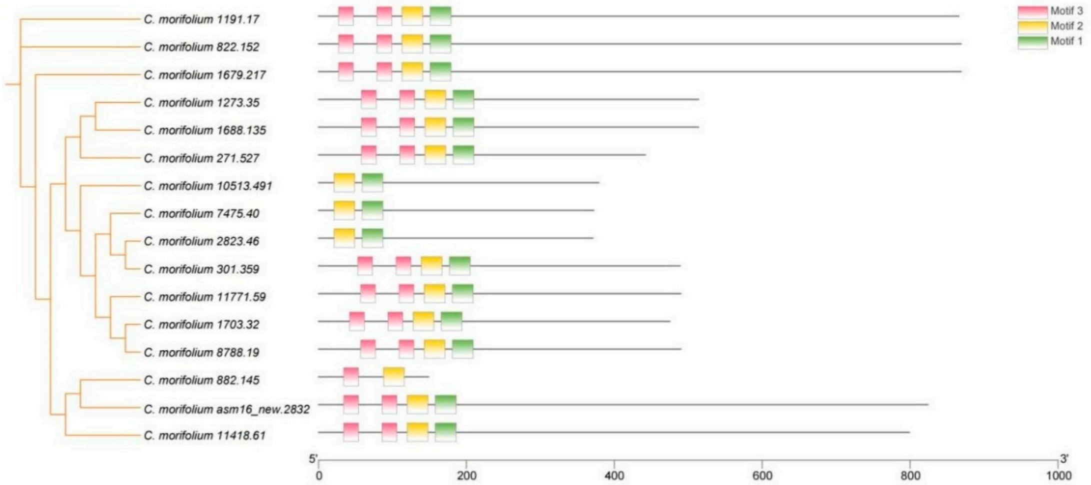

Figure S7. Sequence structure analysis of the 3R-MYB subfamily. 3/20 conserved motifs exist in 3R-MYBs.

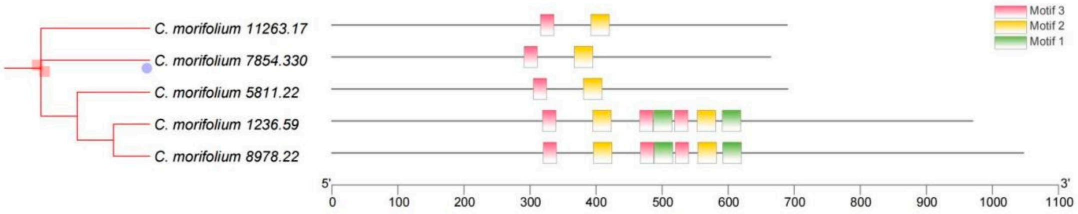

Figure S8. Sequence structure analysis of the 4R-MYB subfamily. 3/20 conserved motifs exist in 4R-MYBs.

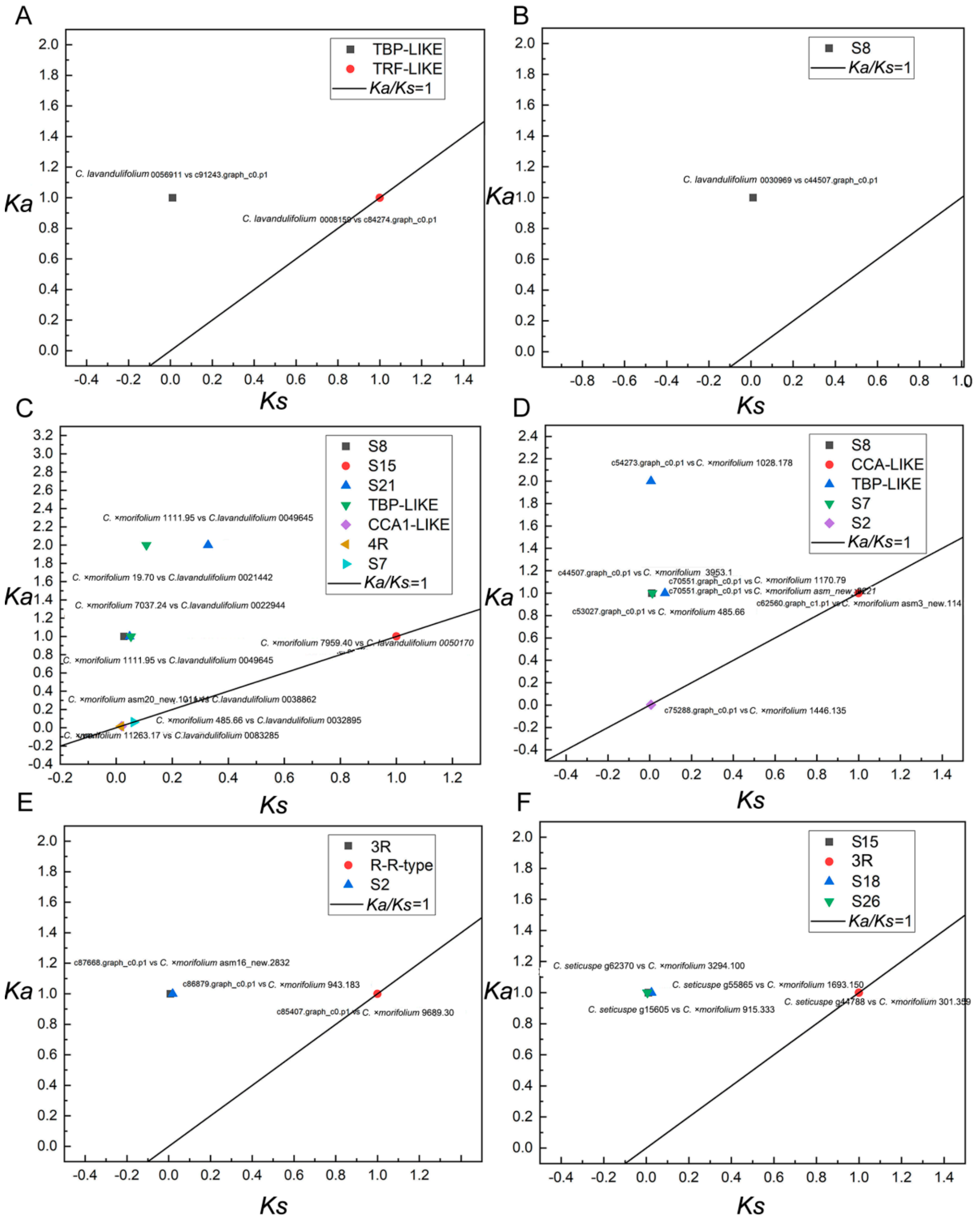

Figure S9. Comparative analysis of homologous *MYB* genes that were subjected to positive selection between *Chrysanthemum* species. (**A**) *C. lavandulifolium* vs. *C. vestitum*; (**B**) *C. lavandulifolium* vs. *C. indicum*; (**C**) *C. lavandulifolium* vs. *C. ×morifolium*; (**D**) *C. indicum* vs. *C. ×morifolium*; (**E**) *C. ×morifolium* vs. *C. vestitum*; (**F**) *C. seticuspe* vs. *C. ×morifolium*. The horizontal and vertical axes represent *Ks* and *Ka* values, respectively. The *Ka/Ks* values of each lineage are marked by different symbols.

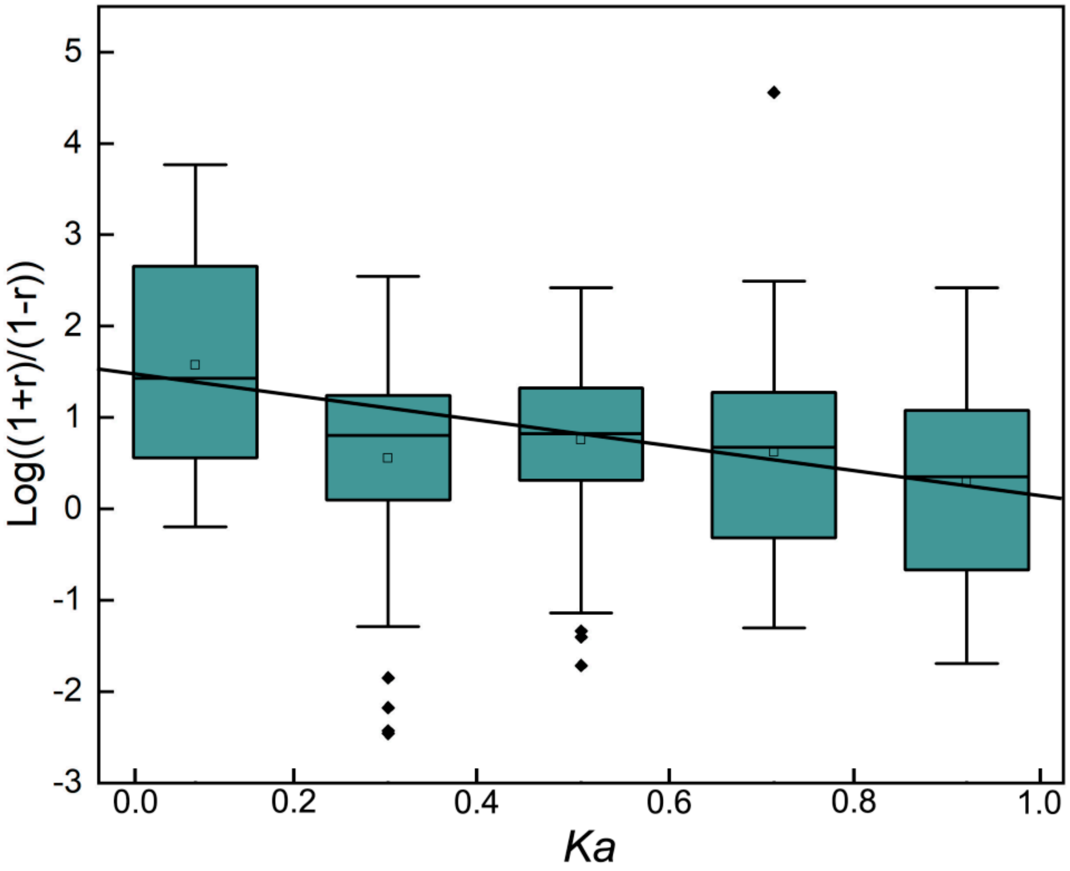

Figure S10. Correlations of expression patterns between homologous genes and *Ka* values in the *MYB* gene family. Fitting function = Log [(1 + *r*) / (1 – *r*)] = 1.5 – 0.25 · *Ka*.

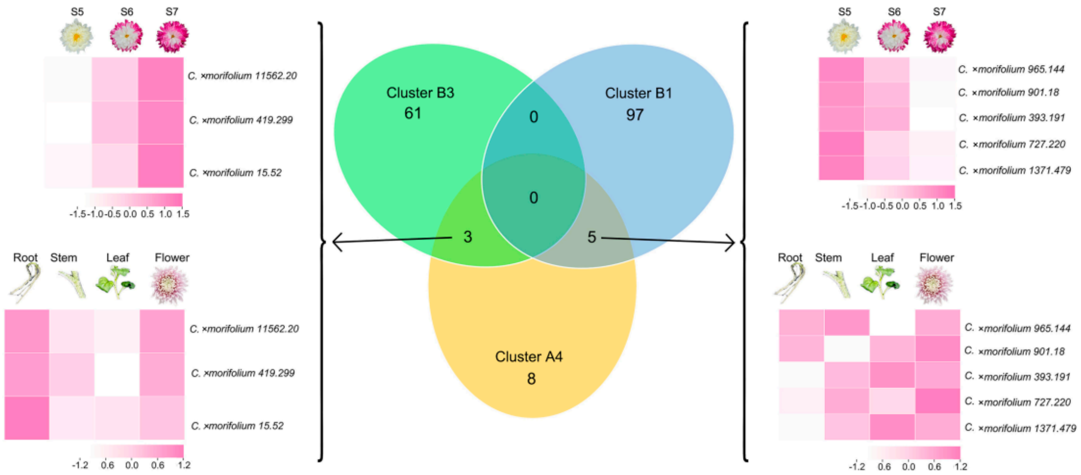

Figure S11. The expression of candidate genes that may regulate anthocyanin biosynthesis in *C. ×morifolium*. S5, S6, and S7 respectively indicate the early, middle, and late stages of anthocyanin accumulation in the capitulum of the cultivated chrysanthemum 'f23'. Numbers in the Venn diagram indicate gene numbers.

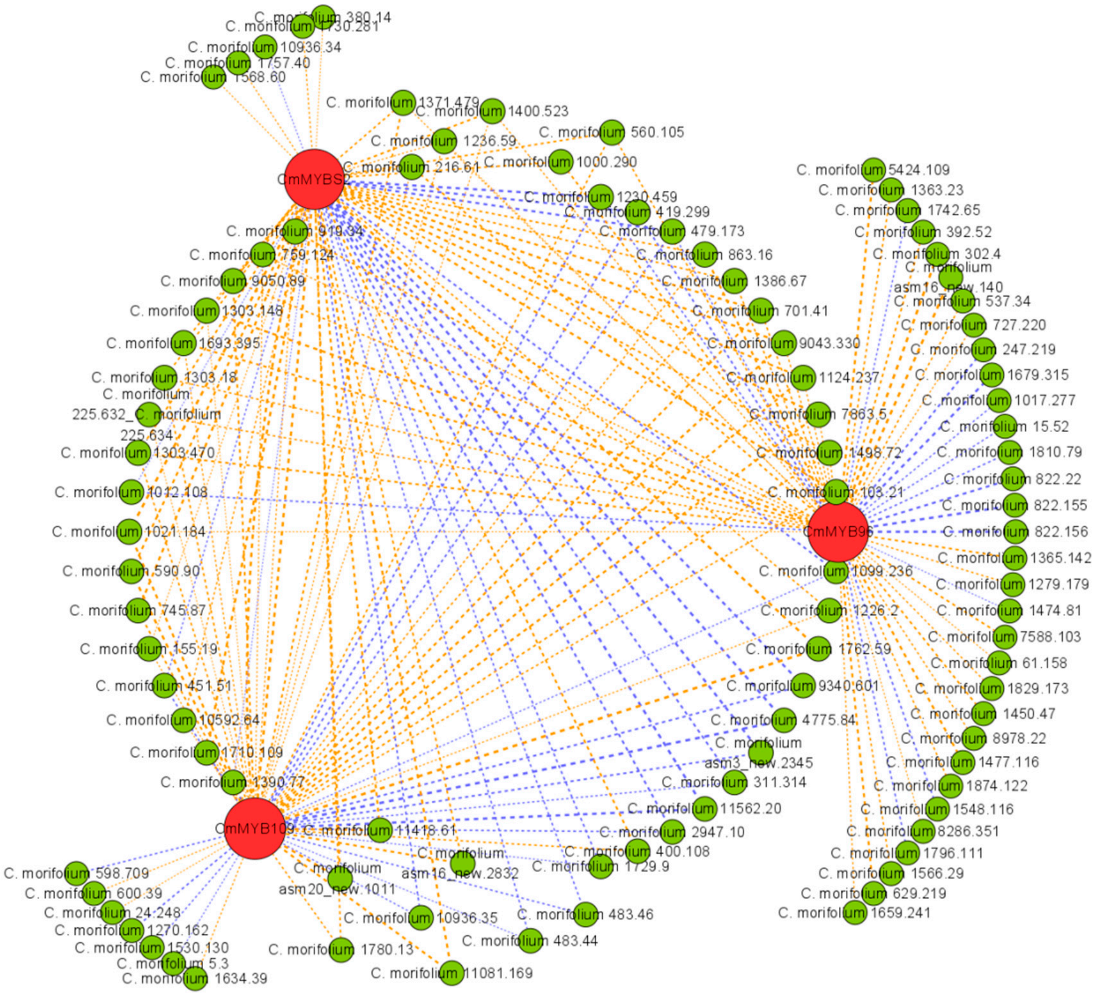

Figure S12. Co-expression networks of *CmMYBS2*, *CmMYB96*, and *CmMYB109* in *C. ×morifolium*. The red nodes indicate bait genes, while the green nodes indicate *MYB* genes that may be related to the biological functions of the bait genes. The blue and orange lines respectively represent negative and positive correlations.
